# Six New Tetraprenylated Alkaloids from the South China Sea Gorgonian *Echinogorgia pseudossapo*

**DOI:** 10.3390/md12020672

**Published:** 2014-01-27

**Authors:** Zhang-Hua Sun, Ying-Hong Cai, Cheng-Qi Fan, Gui-Hua Tang, Hai-Bin Luo, Sheng Yin

**Affiliations:** 1 School of Pharmaceutical Sciences, Sun Yat-sen University, Guangzhou, Guangdong 510006, China; E-Mails: sysuszh@126.com (Z.-H.S.); caiyinghong88@163.com (Y.-H.C.); tanggh5@mail.sysu.edu.cn (G.-H.T.); luohb77@mail.sysu.edu.cn (H.-B.L.); 2 East China Sea Fisheries Research Institute, Chinese Academy of Fishery Sciences, Shanghai 200090, China; E-Mail: chengqifan92@pku.org.cn

**Keywords:** gorgonian, *Echinogorgia pseudossapo*, tetraprenylated alkaloids, phosphodiesterases

## Abstract

Six new tetraprenylated alkaloids, designated as malonganenones L–Q (**1**–**6**), were isolated from the gorgonian *Echinogorgia pseudossapo*, collected in Daya Bay of Guangdong Province, China. The structures of **1**–**6** featuring a methyl group at N-3 and a tetraprenyl chain at N-7 in the hypoxanthine core were established by extensive spectroscopic analyses. Compounds **1**–**6** were tested for their inhibitory activity against the phosphodiesterases (PDEs)-4D, 5A, and 9A, and compounds **1** and **6** exhibited moderate inhibitory activity against PDE4D with IC_50_ values of 8.5 and 20.3 µM, respectively.

## 1. Introduction

**Figure 1:**
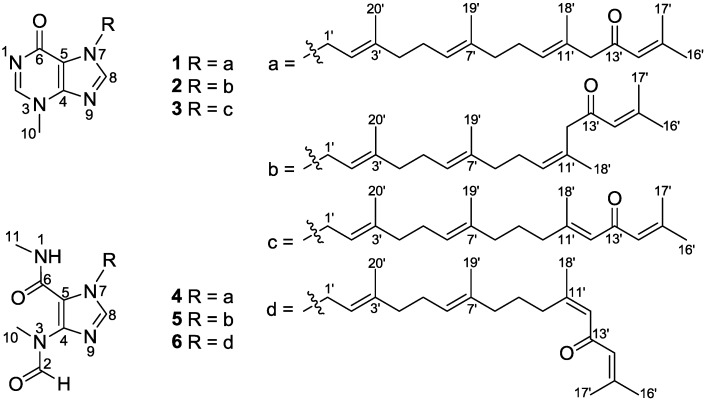
Structures of malonganenones L–Q (**1**–**6**).

Tetraprenylated purine alkaloids and their derivatives are relatively uncommon in nature [1,2]. They are structurally characterized by a methyl group at N-3 and a tetraprenyl chain at N-7 in the hypoxanthine core. Malonganenone A [3], the first typical representative of this series, was isolated from the gorgonian *Leptogorgia gilchristi*, collected in Ponto Malongane, Mozambique in 2006. Until now, only 14 analogues have been reported from marine organisms [3,4,5], some of which exhibited antitumor activity [4,5]. Although the genus *Echinogorgia* is highly prolific in the South China Sea, only a few species of *Echinogorgia* have been chemically investigated, which led to the isolation of a series of metabolites including sterols [6,7,8,9,10,11,12], alkaloids [11,12,13,14], sesquiterpenes [14,15,16], ceramides [17], and coumarins [18]. In our screening program aimed at discovering new biologically active natural products from marine organisms of the South China Sea [19], a fraction of the CH_2_Cl_2_/MeOH extract of *E. pseudosassapo* showed inhibitory activity towards phosphodiesterases (PDEs)-4D, 5A, and 9A. Subsequent chemical investigation resulted in the purification of six new tetraprenylated alkaloids, malonganenones L–Q (**1**–**6**, Figure 1, Supplementary Figures S1–S31). The resulting inhibitory activity screening against PDE4D, PDE5A, and PDE9A showed that compounds **1** and **6** exhibited moderate activities against PDE4D with IC_50_ values of 8.5 and 20.3 µM, respectively. The present report describes the isolation, structure elucidation, and PDEs inhibitory activities of these tetraprenylated alkaloids.

## 2. Results and Discussion

### 2.1. Structural Elucidation of New Compounds

The CH_2_Cl_2_/MeOH (v/v, 1:1) extract of the gorgonian was subjected to chromatography using Sephadex LH-20 followed by silica gel and HPLC separations to yield compounds **1**–**6**.

**Figure 2:**
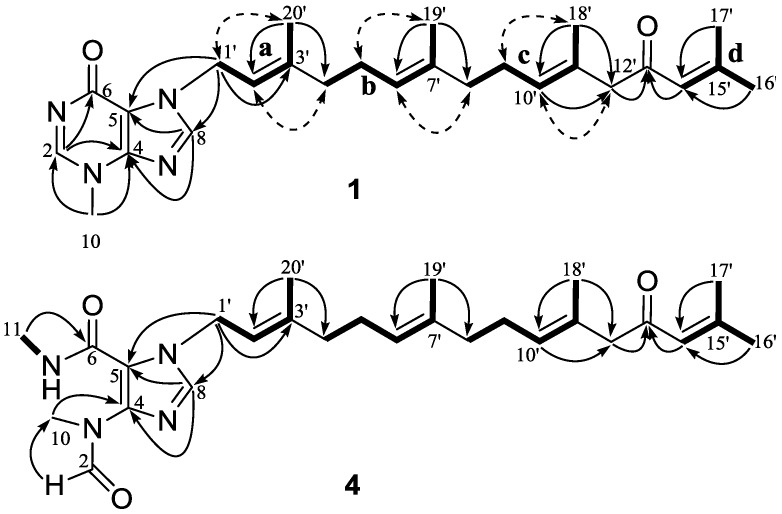
Key ^1^H–^1^H COSY (–), HMBC (→) and NOESY (dashed arrows) correlations for **1** and **4**.

Compound **1**, a colorless oil, exhibited a molecular formula of C_26_H_36_N_4_O_2_ as determined by HRESIMS ([M + Na]^+^, 459.2721, calcd. 459.2736), implying 11 double bond equivalents (DBE). The IR absorption bands at 1709 and 1610 cm^−1^ indicated the presence of two carbonyls. The ^1^H NMR spectrum of **1 **(Table 1) showed signals for two aromatic singlets [δ_H_ 8.26 (H-2) and 7.69 (H-8)], four olefinic protons [δ_H_ 6.07 (H-14′), 5.45 (H-2′), 5.19 (H-10′), and 5.05 (H-6′)], five vinylic methyls [δ_H_ 2.10 (H-17′), 1.84 (H-16′), 1.77 (H-20′), 1.58 (H-18′), and 1.56 (H-19′)], one heteroatom-functionalized methyl [δ_H_ 3.86 (H-10)], and a series of aliphatic methylene multiplets. The ^13^C NMR spectrum of **1 **(Table 2) resolved 26 resonances attributable to five double bonds (δ_C_ 155.5 C, 122.9 CH; 147.3 C, 115.0 C; 143.4 C, 117.6 CH; 135.5 C, 123.5 CH; and 129.6 C, 129.0 CH), two carbonyls (δ_C_ 199.3 and 162.0), two imines (δ_C_ 147.7 and 140.3), five vinylic methyls (δ_C_ 27.7, 20.6, 16.5, 16.4, and 16.0), a *N*-methyl (δ_C_ 35.0), and six sp^3^ methylenes (δ_C_ 55.3, 44.5, 39.4, 39.3, 26.7, and 26.1). As nine of the eleven DBE were accounted for by abovementioned unsaturated functional groups, the remaining two DBE required that **1** was bicyclic. The collective spectroscopic information pointed clearly to a fused diterpene-*N*-methylhypoxanthine structure, which bore a high similarity to that of malonganenone D [4]. The *N*-methylhypoxanthine moiety of **1** was readily identified by comparison of its NMR data with that of malonganenone D, which gave almost identical ^13^C NMR data regarding to this portion. The tetraprenyl side-chain of **1** was deduced from detailed analysis of COSY and HMBC data (Figure 2). ^1^H–^1^H COSY correlations revealed four spin systems: (**a**) H-1′/H-2′/H-3′/H_3_-20′; (**b**) H-4′/H-5′/H-6′/H-7′/H_3_-19′; (**c**) H-8′/H-9′/H-10′/H-11′/H_3_-18′; and (**d**) H-14′/H-15′/H-16′ or H-17′. The connections from **a** to **c** were achieved by HMBC correlations of H-20′/C-4′ and H-19′/C-8′. The fragments **c** and **d** were linked via a methylene and a ketone (C-13) by HMBC correlations of H-18′/C-12′, H-12′/C-13′, and H-14′/C-13′. The geometries of the three olefins in the tetraprenyl side-chain were established from analysis of both ^13^C chemical shift and NOE data. The ^13^C chemical shifts of the vinylic methyls of C-18′, C-19′, and C-20′ (δ_C_ 16.4, 16.0, and 16.5, respectively) suggested the *E* geometries for ∆^10′^, ∆^6′^, and ∆^2′^ as the vinyl methyl corresponding to *Z* geometry are known to resonate at around 25 ppm [4]. This was further supported by NOE correlations (Figure 2) of H-9′/H-18′ and H-10′/H-12′, H-5′/H-19′ and H-6′/H-8′, and H-1′/H-20′ and H-2′/H-4′, respectively. Finally, the tetraprenyl side-chain was attached to N-7 by HMBC correlations from H-1′ to C-5 and C-8. Thus, compound **1** was determined as depicted and given the trivial name malonganenone L.

**Table 1:** ^1^H NMR spectroscopic data for malonganenones L–Q (**1**–**6**) (δ in ppm, *J* in Hz).

No.	1 ^a^	2 ^a^	3 ^a^	4 ^b^	5 ^b^	6 ^b^
1				7.23, brs		7.22, brs
2	8.26, br s	8.57, br s	8.62, br s	8.23, s	8.23, s	8.23, s
8	7.69, s	7.73, s	7.73, s	7.59, s	7.59, s	7.57, s
10	3.86, s	3.94, s	3.95, s	3.13, s	3.13, s	3.13, s
11				2.84, s	2.84, s	2.84, d (4.7)
1′	5.07, d (7.3)	5.09, d (7.2)	5.08, d (7.4)	4.90, d (7.0)	4.90, d (7.0)	4.89, d (7.1)
2′	5.45, t (7.0)	5.47, t (6.9)	5.47, t (6.9)	5.40, t (7.1)	5.41, t (7.0)	5.39, t (7.0)
4′	2.08, m	2.11, m	2.11, m	2.07, m	2.08, m	2.09, m
5′	2.09, m	2.11, m	2.12, m	2.12, m	2.13, m	2.13, m
6′	5.05, m	5.08, m	5.07, m	5.14, t (6.2)	5.14, t (6.1)	5.14, t (6.3)
8′	1.98, m	1.99, m	1.97, m	2.01, m	2.01, m	2.02, m
9′	2.04, m	2.07, m	1.55, m	2.12, m	2.12, m	1.56, m
10′	5.19, t (6.4)	5.32, t (6.6)	2.06, m	5.26, t (6.8)	5.30, t (6.3)	2.55, br t, (7.9)
12′	2.99, s	3.09, s	6.00, br s	3.01, s	3.12, s	6.08, br s
14′	6.07, br s	6.06, br s	6.04, br s	6.17, br s	6.15, br s	6.10, br s
16′	1.84, s	1.87, s	1.87, d (0.9)	1.86, d (1.1)	1.87, d (1.1)	1.86, d (1.0)
17′	2.10, s	2.13, s	2.13, d (1.0)	2.09, d (1.1)	2.09, d (1.0)	2.12, d (1.0)
18′	1.58, s	1.69, s	2.15, d (1.2)	1.61, s	1.66, d (1.2)	1.87, d (1.3)
19′	1.56, s	1.58, s	1.58, s	1.59, s	1.60, s	1.62, s
20′	1.77, s	1.79, s	1.80, s	1.79, s	1.79, s	1.79, s

^a^ Measured at 400 MHz in CDCl_3_; ^b^ Measured at 400 MHz in Acetone-*d*_6_.

**Table 2:** ^13^C NMR spectroscopic data for malonganenones L–Q (**1**–**6**) (δ in ppm).

No.	1 ^a^	2 ^a^	3 ^a^	4 ^b^	5 ^b^	6 ^b^
2	147.7	148.1	148.1	163.3	163.3	163.3
4	147.3	147.2	147.2	141.8	141.8	141.8
5	115.0	115.2	115.2	118.2	118.2	118.2
6	162.0	160.4	160.3	160.9	160.9	161.0
8	140.3	140.7	140.8	137.6	137.6	137.6
10	35.0	35.5	35.5	31.9	31.9	31.9
11				26.1	26.1	26.3
1′	44.5	44.6	44.7	45.2	45.2	45.2
2′	117.6	117.3	117.4	120.5	120.5	120.4
3′	143.4	143.7	143.7	142.0	142.0	142.0
4′	39.4	39.5	39.4	40.1	40.2	40.2
5′	26.1	26.1	26.1	27.0	26.9	26.9
6′	123.5	123.6	123.9	124.8	124.8	124.8
7′	135.5	135.5	135.4	135.8	135.8	136.0
8′	39.3	39.5	39.1	40.1	40.3	40.6
9′	26.7	26.9	25.8	27.4	27.7	27.2
10′	129.0	128.2	40.8	129.4	128.7	33.8
11′	129.6	129.2	157.8	130.7	130.2	158.6
12′	55.3	47.9	125.7	55.8	48.3	126.9
13′	199.3	198.6	191.7	198.8	198.1	191.0
14′	122.9	123.0	126.3	123.7	124.0	127.0
15′	155.5	155.8	154.2	155.0	155.3	154.2
16′	27.7	27.7	27.7	27.5	27.5	27.6
17′	20.6	20.7	20.5	20.5	20.5	20.4
18′	16.4	24.1	19.1	16.5	24.4	25.5
19′	16.0	16.0	15.9	16.1	16.1	16.0
20′	16.5	16.6	16.6	16.5	16.5	16.5

^a^ Measured at 100 MHz in CDCl_3_; ^b^ Measured at 100 MHz in Acetone-*d*_6_.

Compound **2** exhibited the same molecular formula of C_26_H_36_N_4_O_2_ as **1** on the basis of the HRESIMS data ([M + Na]^+^, 459.2725, calcd. 459.2736). The NMR spectroscopic data of **2** (Table 1 and Table 2) was very similar to that of **1**. In comparison with **1**, the ^13^C NMR spectroscopic data for **2** differed significantly about ∆^10′^ moiety, with the upfield-shifted carbon at C-12′ and the downfield-shifted vinyl methyl at C-18′ (δ_C_ 55.3 and 16.3 in **1**; δ_C_ 47.9 and 24.1 in **2**, respectively). This indicated that ∆^10′^ in **2** adopts a *Z* configuration. Similar ^13^C NMR changes were also reported in malonganenone I [5], which possessed the same *Z* configuration of ∆^10′^ as **2**. Thus, compound **2** was determined as depicted and named malonganenone M.

Compound **3** had a molecular formula of C_26_H_36_N_4_O_2_ as established by HRESIMS data. The ^1^H and ^13^C NMR data of **3 **(Table 1 and Table 2) showed high similarity to those of **1** except that the ∆^10′^ double bond in **1** was migrated to ∆^11′^, forming a conjugated system with the C-13′ carbonyl. This was suggested by the significant downfield-shifted carbon at C-11′ and the upfield-shifted carbon at C-13′ as compared with those of **1** (δ_C_ 129.6 and 199.3 in **1**; δ_C_ 157.8 and 191.7 in **3**, respectively), and by the presence of a singlet olefinic signal (δ_H_ 6.00, H-12′) in the ^1^H NMR spectra of **3** instead of a triplet olefinic signal (δ_H_ 5.19, t, *J* = 6.4 Hz, H-10′) in **1**. The configuration of ∆^11′^ in **3** was established to be *E* by the characteristic chemical shift of the vinylic methyl at C-18′ (δ_C_ 19.1) and by comparison of its NMR data with those of reported. Therefore, the structure of compound **3** was determined as depicted and given the trivial name malonganenone N.

Compound **4** exhibited an [M − H]^−^ ion at *m*/*z* 467.3021 (calcd. for C_27_H_39_N_4_O_3_, 467.3022), suggesting the molecular formula C_27_H_40_N_4_O_3_ (ten DBE). The ^1^H and ^13^C NMR spectra of **4** (Table 1 and Table 2) bore a resemblance to those of **1**, with the notable differences occurring in the hypoxanthine core. The NMR spectra of **4** showed the presence of an *N*-methylamide (δ_H_ 2.84, H-11; δ_C_ 26.1, C-11) and an *N*-methylformamide (δ_H_ 8.23, H-2 and 3.13, H-10; δ_C_ 163.3, C-2 and 31.9, C-10) groups, which were identical to those previously reported in malonganenones B, F, and G, indicating that **4** possessed the same trisubstituted imidazole ring. This was further supported by HMBC correlations (Figure 2) of H_3_-11/C-6, H_3_-10/C-4, and H-2/C-10. Thus, the structure of compound **4** was determined as depicted and given the trivial name malonganenone O.

Compound **5** had a molecular formula C_27_H_40_N_4_O_3_ by analysis of the HRESIMS data. Comparing the NMR data (Table 1 and Table 2) of **5** and **4**, it appeared that the former had a *Z* configuration of ∆^10′^ instead of an *E* configuration of ∆^10′^ in **4**. This was suggested by the upfield-shifted carbon at C-12′ and the downfield-shifted vinyl methyl at C-18′ (δ_C_ 48.3 and 24.4 in **5**; δ_C_ 55.8 and 16.5 in **4**, respectively). Thus, the structure of compound **5** was determined as depicted and given the trivial name malonganenone P.

The molecular formula of compound **6** was established as C_27_H_40_N_4_O_3_ by HRESIMS. The NMR data of **6** (Table 1 and Table 2) showed high similarity to that of **4** except that the ∆^10′^ double bond in **6 **was migrated to ∆^11′^, forming a conjugated system with the C-13′ carbonyl. This was further suggested by the significant downfield-shifted carbon at C-11′ and the upfield-shifted carbon at C-13′ as compared with those of **4** (δ_C_ 130.7 and 198.8 in **4**; δ_C_ 158.6 and 191.0 in **6**, respectively), and by the presence of a singlet olefinic signal (δ_H_ 6.08, H-12′) in the ^1^H NMR spectra of **6** instead of a triplet olefinic signal (δ_H_ 5.26, t, *J* = 6.8 Hz, H-10′) in **4**. The characteristic chemical shift of the vinylic methyl at C-18′ (δ_C_ 25.5) indicated the *Z* configuration of ∆^11′^ in **6**. Thus, the structure of **6** was determined as depicted and given the trivial name malonganenone Q.

### 2.2. *In Vitro* Inhibitory Activity Screening against PDEs

**Figure 3:**
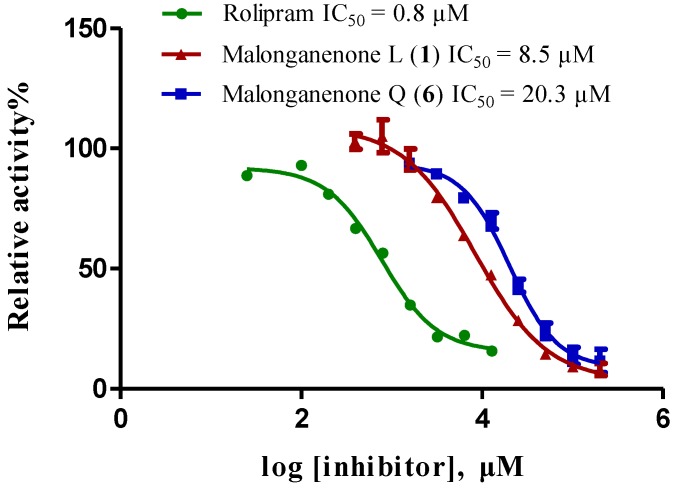
Inhibition of phosphodiesterase-4D by compounds **1** and **6** (rolipram as positive control).

Compounds **1**–**6** were screened for their inhibitory activities against PDE4D, PDE5A, and PDE9A by using our previously reported methods [20,21,22,23]. As shown in Table 3, all compounds exhibited inhibition at 50 µM against PDE4D with degree of inhibition from 72% to 85%, while displaying weaker activities against PDE5A and PDE9A. The two most active compounds, **1** and **6**, were selected to test for the half maximal inhibitory concentration (IC_50_), which gave IC_50_ values of 8.5 and 20.3 µM, respectively (Figure 3).

**Table 3:** Inhibitory activities of compounds **1**–**6** at 5 µM and 50 µM towards PDE4D, PDE5A and PDE9A.

Compound	Inhibition (%) of Compounds at 50 µM				Inhibition (%) of Compounds at 5 µM		
	PDE4D	PDE5A	PDE9A		PDE4D	PDE5A	PDE9A
**1**	85	53	18		17	11	<10
**2**	72	32	15		<10	<10	<10
**3**	81	35	27		17	<10	10
**4**	79	38	<10		18	<10	<10
**5**	75	36	11		14	<10	<10
**6**	85	38	15		18	<10	<10

## 3. Experimental Section

### 3.1. General Experimental Procedures

UV spectra were recorded on a Shimadzu UV-2450 spectrophotometer. IR spectra were determined on a Bruker Tensor 37 infrared spectrophotometer with KBr disks. NMR spectra were measured on a Bruker AM-400 spectrometer at 25 °C. ESIMS and HRESIMS were carried out on a Finnigan LC Q^DECA^ instrument. A Shimadzu LC-20 AT equipped with an SPD-M20A PDA detector was used for HPLC, a YMC-pack ODS-A column (250 × 10 mm, 5 µm, 12 nm) and a chiral column (Phenomenex Lux, cellulose-2, 250 × 10 mm, 5 µm) was used for semipreparative HPLC separation. Silica gel (300–400 mesh, Qingdao Marine Chemical Co., Ltd., Qingdao, Shandong, China), C_18_ reversed-phase (Rp-C_18_) silica gel (12 nm, 50 µm, YMC Co., Ltd., Kyoto, Japan), Sephadex LH-20 gel (Amersham Biosciences, Piscataway, NJ, USA), used for column chromatography (CC). All solvents used were of analytical grade (Guangzhou Chemical Reagents Company, Ltd., Guangzhou, Guangdong, China).

### 3.2. Animal Material

The gorgonian *E. pseudosassapo* was collected at a depth of 18–25 m in 29 July 2012 in Daya Bay of Guangdong Province, China and frozen immediately after collection, and were identified by one of the authors (Cheng-Qi Fan). A voucher specimen (accession number: LSH201207) has been deposited at the School of Pharmaceutical Sciences, Sun Yat-sen University, China.

### 3.3. Extraction and Isolation

Specimens of *E. pseudosassapo* (550 g, wet weight) were extracted with CH_2_Cl_2_/MeOH (1:1, 3 × 1 L) at room temperature (rt) to give 13.7 g of crude extract. The crude extract was subjected to silica gel column chromatography eluted with a CH_2_Cl_2_/MeOH gradient (9:1→1:9) to afford five fractions (Fr. I–V). Fr. III (1.4 g) was chromatographed over Sephadex LH-20 (CH_2_Cl_2_/MeOH, v/v, 1:1), followed by Rp-C_18_ silica gel eluted with a CH_3_CN/H_2_O gradient (5:5→10:0) to obtain four sub-fractions (Fr. IIIa–IIId). Fr. IIIb was further separated by HPLC equipped with a chiral column (CH_3_CN, 3 mL/min) to afford **1** (17 mg, *t*_R_ 17 min) and **2** (4.9 mg, *t*_R_ 20 min). Fr. IIId was purified by repeating the HPLC conditions described above to yield **4** (11 mg, *t*_R_ 23 min) and **5** (3.7 mg, *t*_R_ 27 min). Fr. IIIc was chromatographed by HPLC equipped with an ODS-A column (CH_3_CN/H_2_O, 90:10, 3 mL/min) to afford **3** (5.2 mg, *t*_R_ 15 min) and **6** (5.1 mg, *t*_R_ 20min).

**Malonganenone L **(**1**): colorless oil; UV (MeOH) λ_max_ (log ε) 211 (4.42), 225 (4.38), 253 (4.31) nm; IR ν_max_ 3145, 1709, 1610, 1464, 1250, 1128, 1060 cm^−1^; ^1^H and ^13^C NMR, see Table 1 and Table 2; HRESIMS [M + Na]^+^ *m*/*z* 459.2721 (calcd. for C_26_H_36_N_4_O_2_Na, 459.2736).

**Malonganenone M **(**2**): colorless oil; UV (MeOH) λ_max_ (log ε) 209 (4.22), 225 (4.17), 254 (4.06) nm; IR ν_max_ 3011, 1714, 1607, 1458, 1240, 1123, 1046 cm^−1^; ^1^H and ^13^C NMR, see Table 1 and Table 2; HRESIMS [M + Na]^+^ *m*/*z* 459.2725 (calcd. for C_26_H_36_N_4_O_2_Na, 459.2736).

**Malonganenone N **(**3**): colorless oil; UV (MeOH) λ_max_ (log ε) 208 (3.68), 223 (3.60), 254 (3.16) nm; IR ν_max_ 2937, 1732, 1627, 1439, 1379, 1215, 1136, 1039 cm^−1^; ^1^H and ^13^C NMR, see Table 1 and Table 2; HRESIMS [M + Na]^+^ *m*/*z* 459.2725 (calcd. for C_26_H_36_N_4_O_2_Na, 459.2736).

**Malonganenone O **(**4**): colorless oil; UV (MeOH) λ_max_ (log ε) 211 (4.39), 249 (4.22) nm; IR ν_max_ 2932, 1744, 1654, 1514, 1445, 1218, 1127, 1033 cm^−1^; ^1^H and ^13^C NMR, see Table 1 and Table 2; HRESIMS [M − H]^−^ *m*/*z* 467.3021 (calcd. for C_27_H_39_N_4_O_3_, 467.3022).

**Malonganenone P **(**5**): colorless oil; UV (MeOH) λ_max_ (log ε) 210 (4.27), 254 (4.02) nm; IR ν_max_ 2933, 1674, 1621, 1437, 1220, 1128, 1018 cm^−1^; ^1^H and ^13^C NMR, see Table 1 and Table 2; HRESIMS [M + Na]^+^ *m*/*z* 491.2994 (calcd. for C_27_H_40_N_4_O_3_Na, 491.2998).

**Malonganenone Q **(**6**): colorless oil; UV (MeOH) λ_max_ (log ε) 212 (4.52), 257 (4.46) nm; IR ν_max_ 2932, 1664, 1623, 1532, 1443, 1224, 1057 cm^−1^; ^1^H and ^13^C NMR, see Table 1 and Table 2; HRESIMS [M + Na]^+^ *m*/*z* 491.2994 (calcd. for C_27_H_40_N_4_O_3_Na, 491.2998).

## 4. Conclusions

In our continuing investigation on the chemical constituents of marine invertebrates collected from the South China Sea, six new tetraprenylated alkaloids, designated as malonganenones L–Q (**1**–**6**), were isolated from the gorgonian *Echinogorgia pseudossapo*. The structures of **1**–**6** featuring a methyl group at N-3 and a tetraprenyl chain at N-7 in the hypoxanthine core were established by extensive spectroscopic analyses. Compounds **1**–**6** were tested for their inhibitory activity against the phosphodiesterases (PDEs)-4D, 5A, and 9A, and compounds **1** and **6** exhibited moderate inhibitory activity against PDE4D with IC_50_ values of 8.5 and 20.3 µM, respectively. Phosphodiesterase-4 (PDE4), which specifically catalyzes the hydrolysis of cyclic adenosine monophosphate (cAMP), is a therapeutic target of high interest for central nervous system (CNS), inflammatory, and respiratory diseases [24]. Natural PDE4 inhibitors are very rare. To the best of our knowledge, this is the first investigation of this group of compounds on the inhibitory activity of the phosphodiesterases.

## Supplementary Files

Supplementary File 1 Supplementary Information (PDF, 2550 KB)
