# Supplementary material for: Six New Tetraprenylated Alkaloids from the South China Sea Gorgonian Echinogorgia pseudossapo
Source: Mar Drugs. 2014 Jan 27;12(2):672–81. doi: 10.3390/md12020672 (PMC3944508; doi:10.3390/md12020672)
Supplement: Supplementary File 1 — Supplementary Information (PDF, 2550 KB) [file marinedrugs-12-00672-s001.pdf]

Supplementary Information

Figure S1. ^{1}H NMR spectrum of Malonganenone L (**1**).

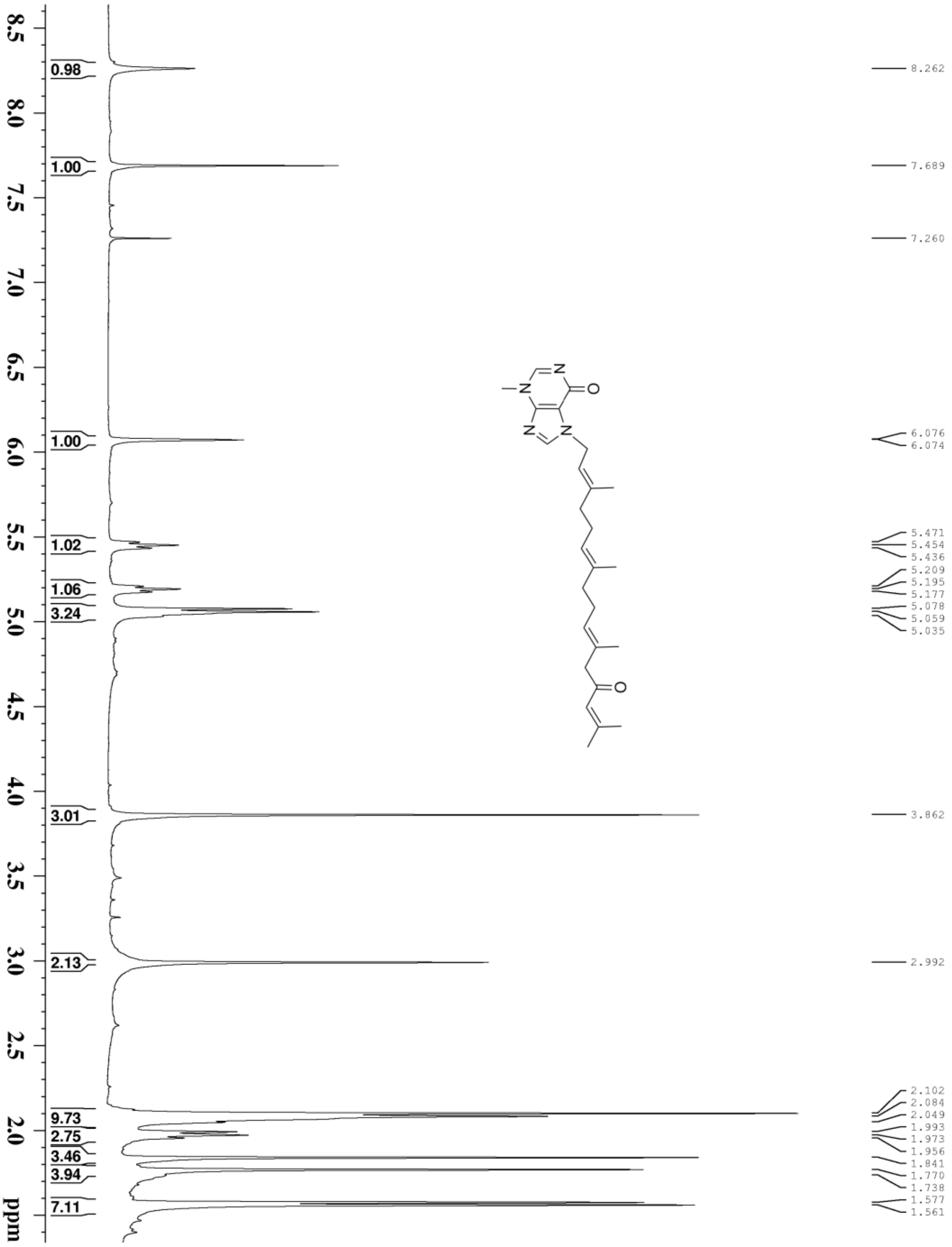

Figure S2. ^{13}C NMR spectrum of Malonganenone L (**1**).

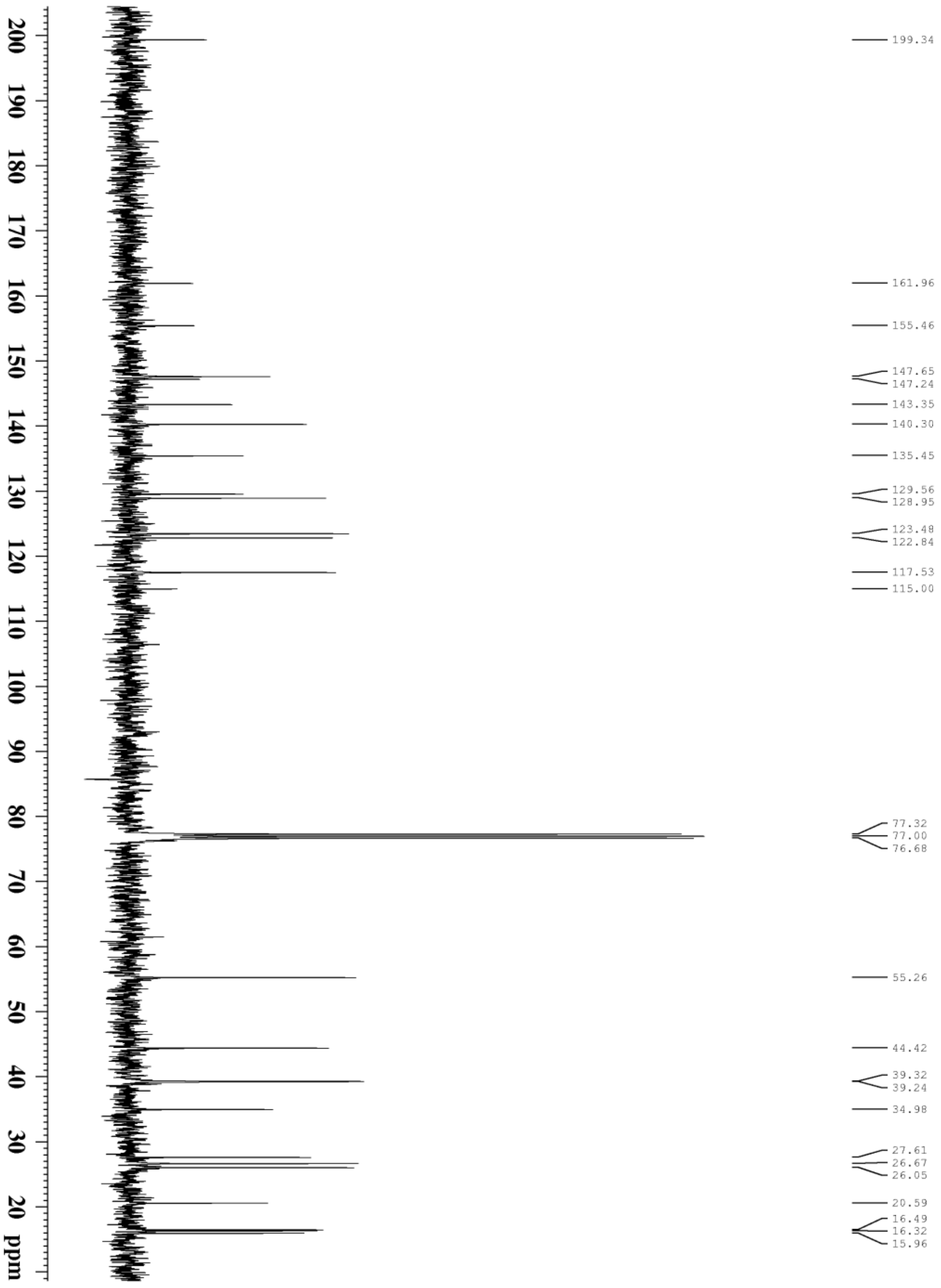

Figure S3. HSQC Spectrum of Malonganenone L (**1**).

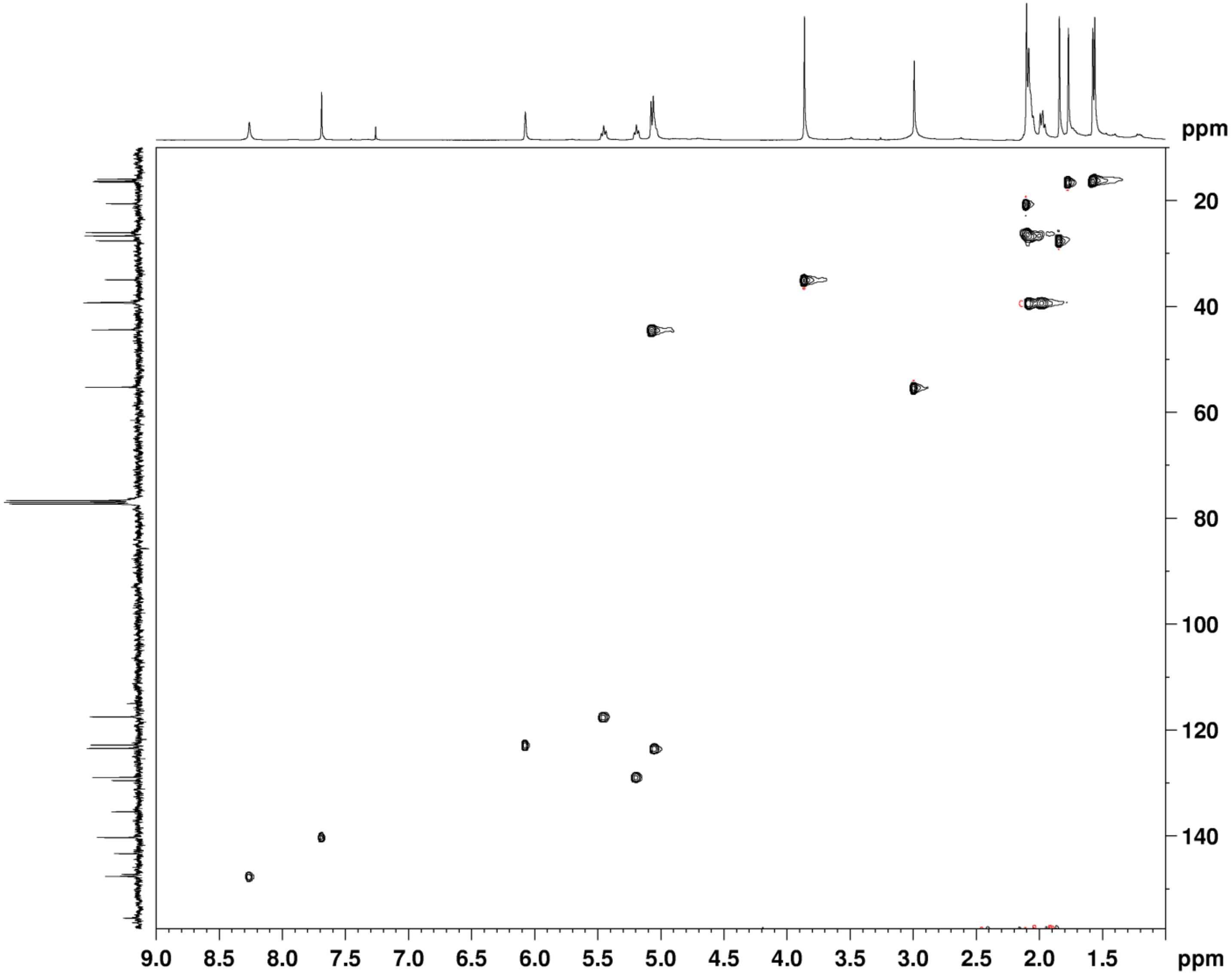

Figure S4. ^{1}H–^{1}H COSY spectrum of Malonganenone L (**1**).

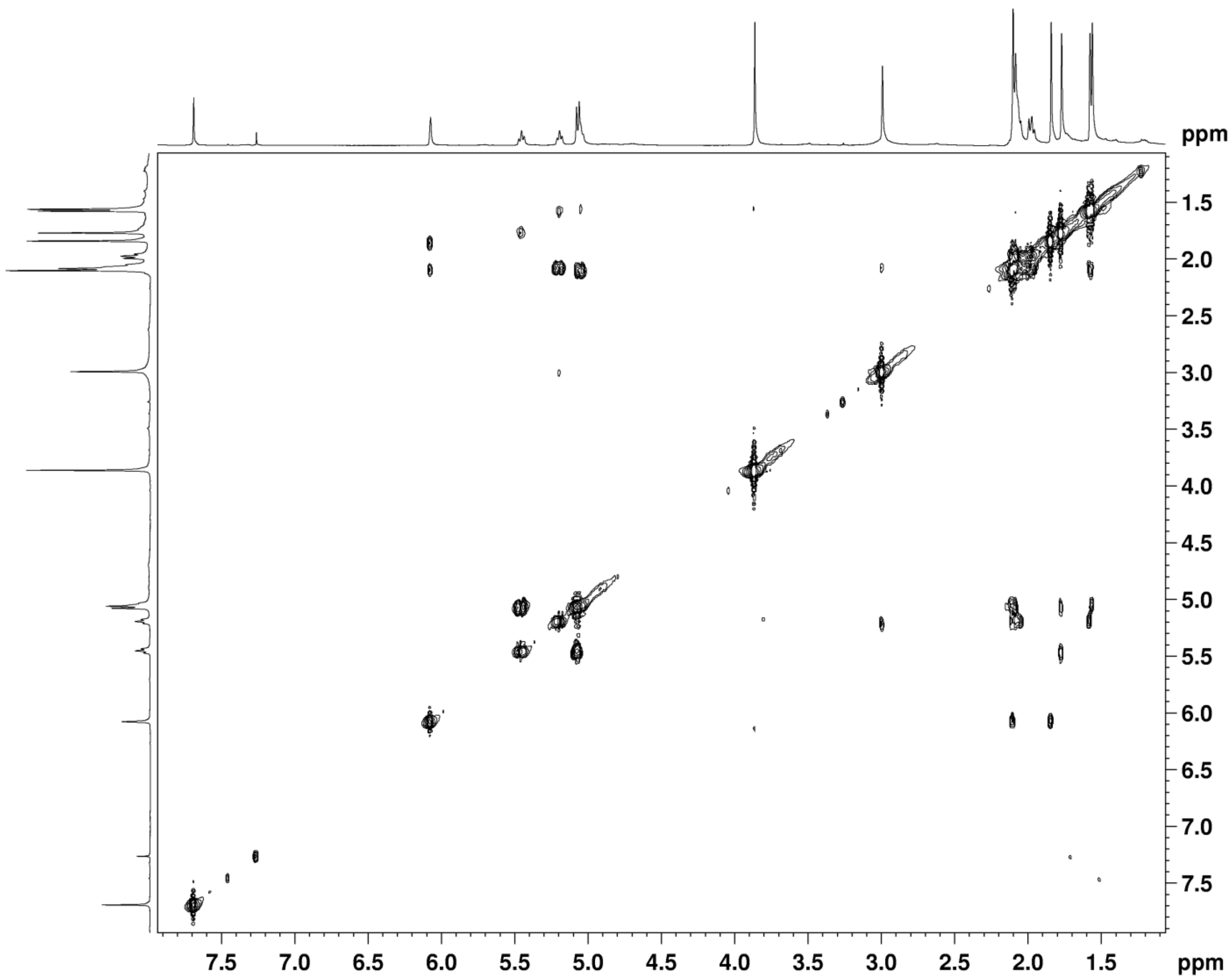

Figure S5. HMBC spectrum of Malonganenone L (**1**).

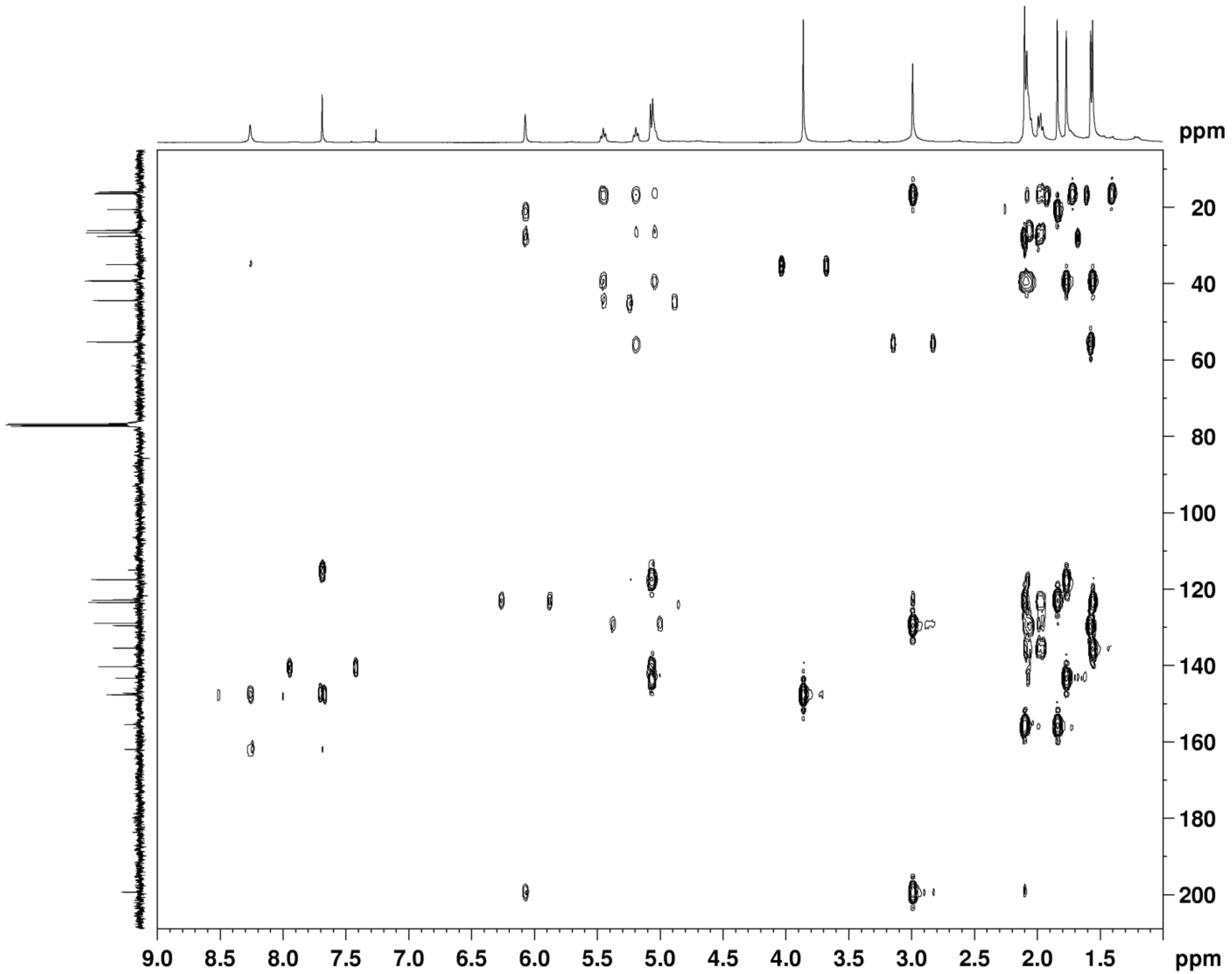

Figure S6. NOESY spectrum of Malonganenone L (**1**).

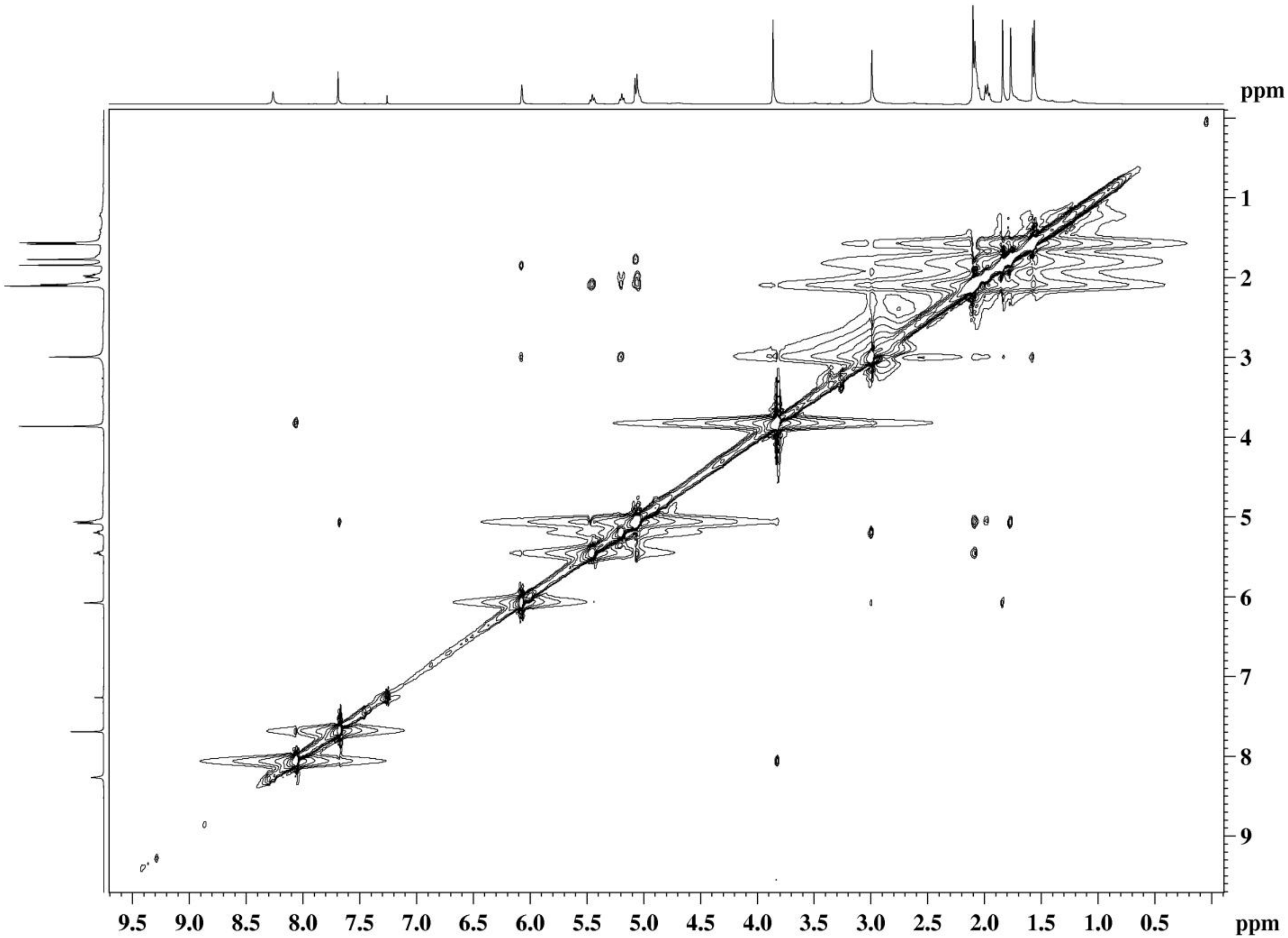

Figure S7. ^{1}H NMR spectrum of Malonganenone M (**2**).

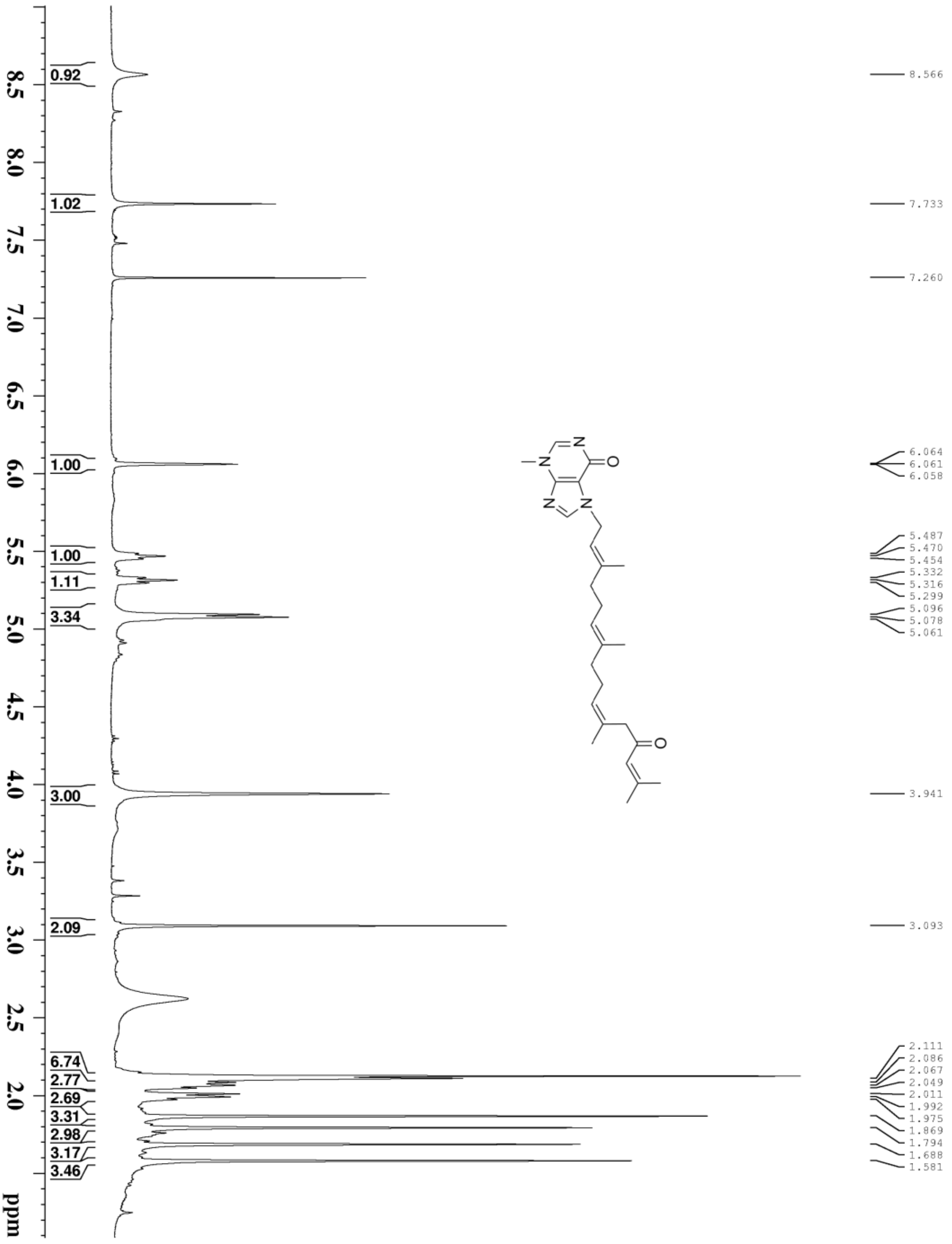

Figure S8. ^{13}C NMR spectrum of Malonganenone M (**2**).

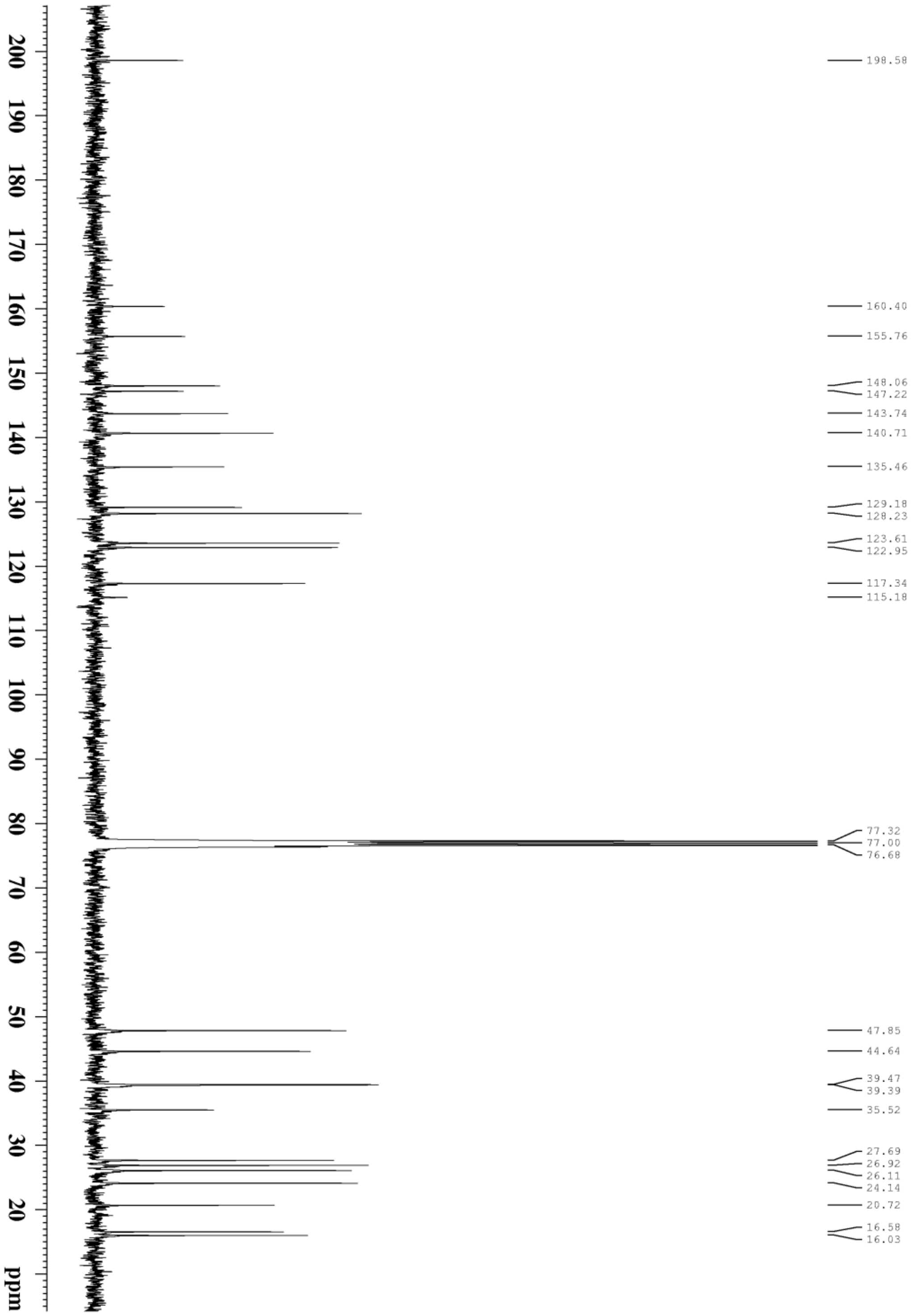

Figure S9. HSQC Spectrum of Malonganenone M (**2**).

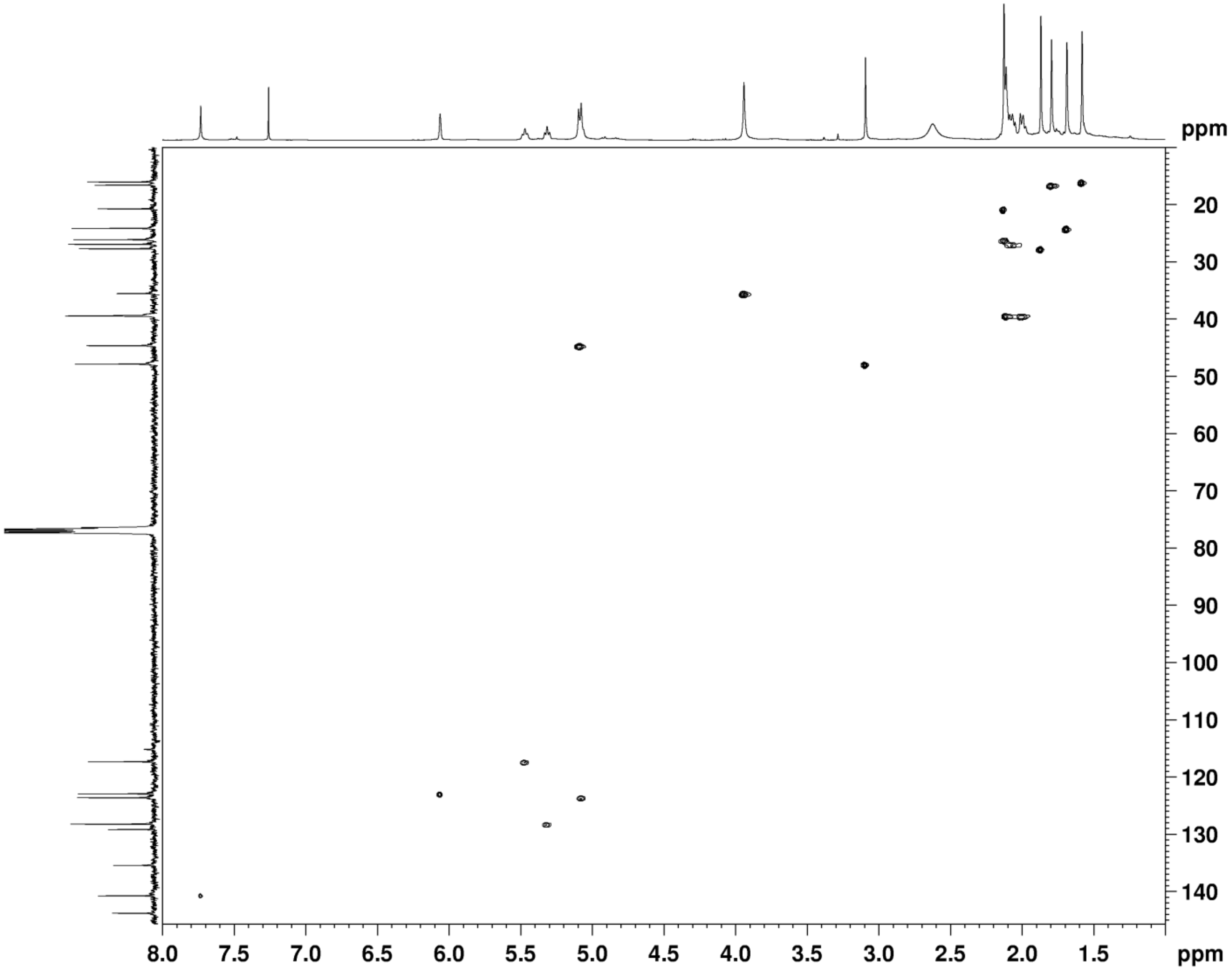

Figure S10. ^1H–^1H COSY spectrum of Malonganenone M (**2**).

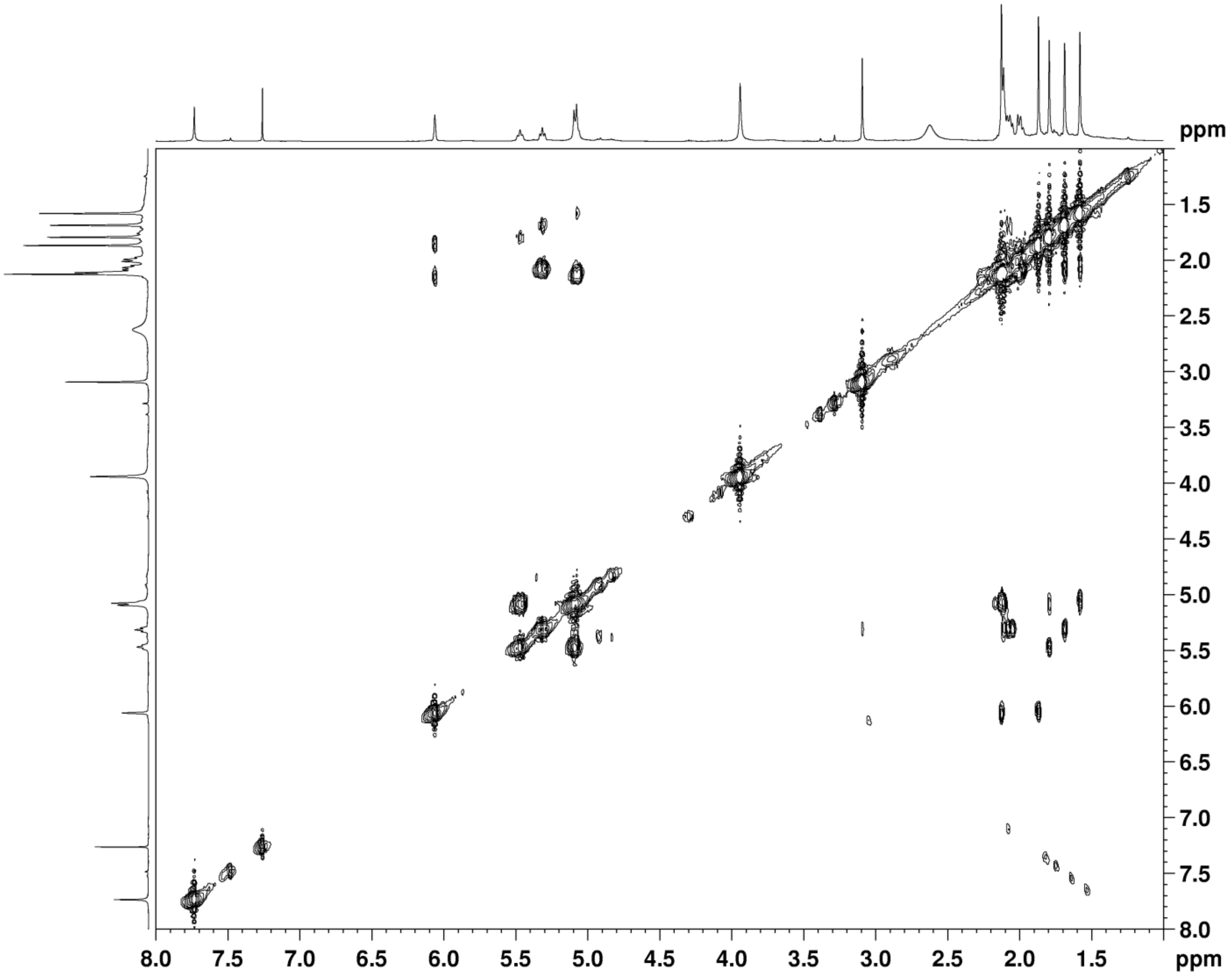

Figure S11. HMBC spectrum of Malonganenone M (**2**).

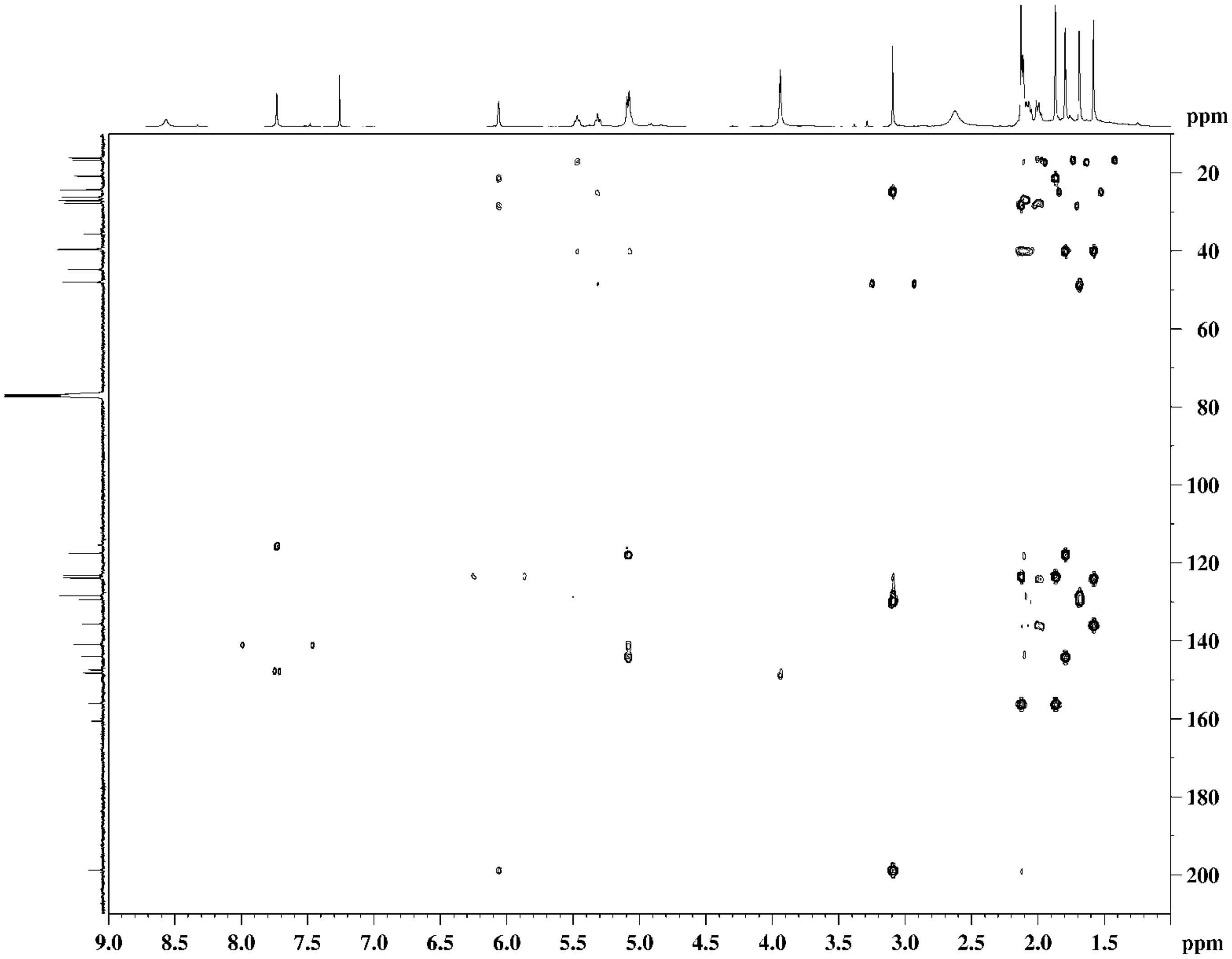

Figure S12. ^{1}H NMR spectrum of Malonganenone N (**3**).

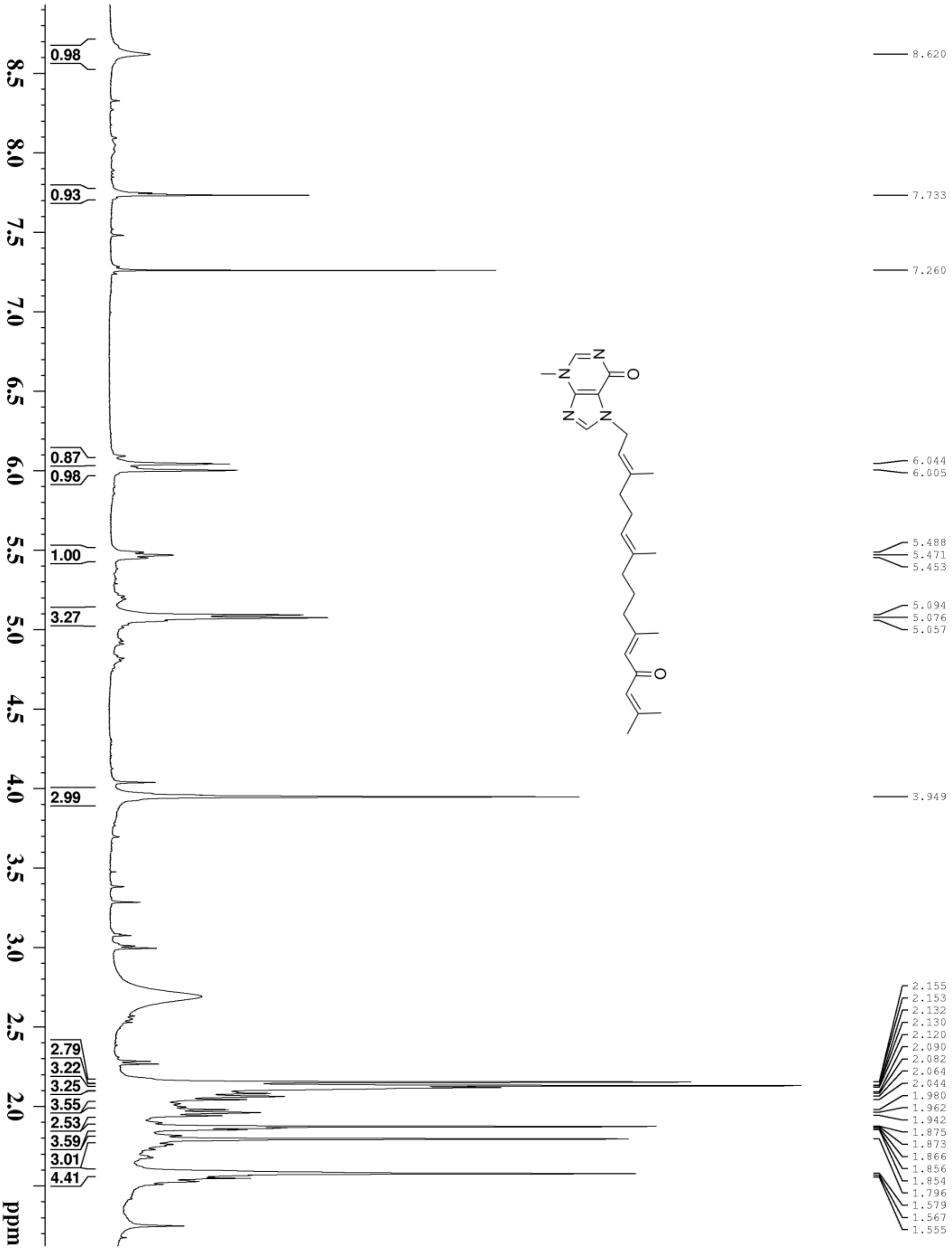

Figure S13. ^{13}C NMR spectrum of Malonganenone N (**3**).

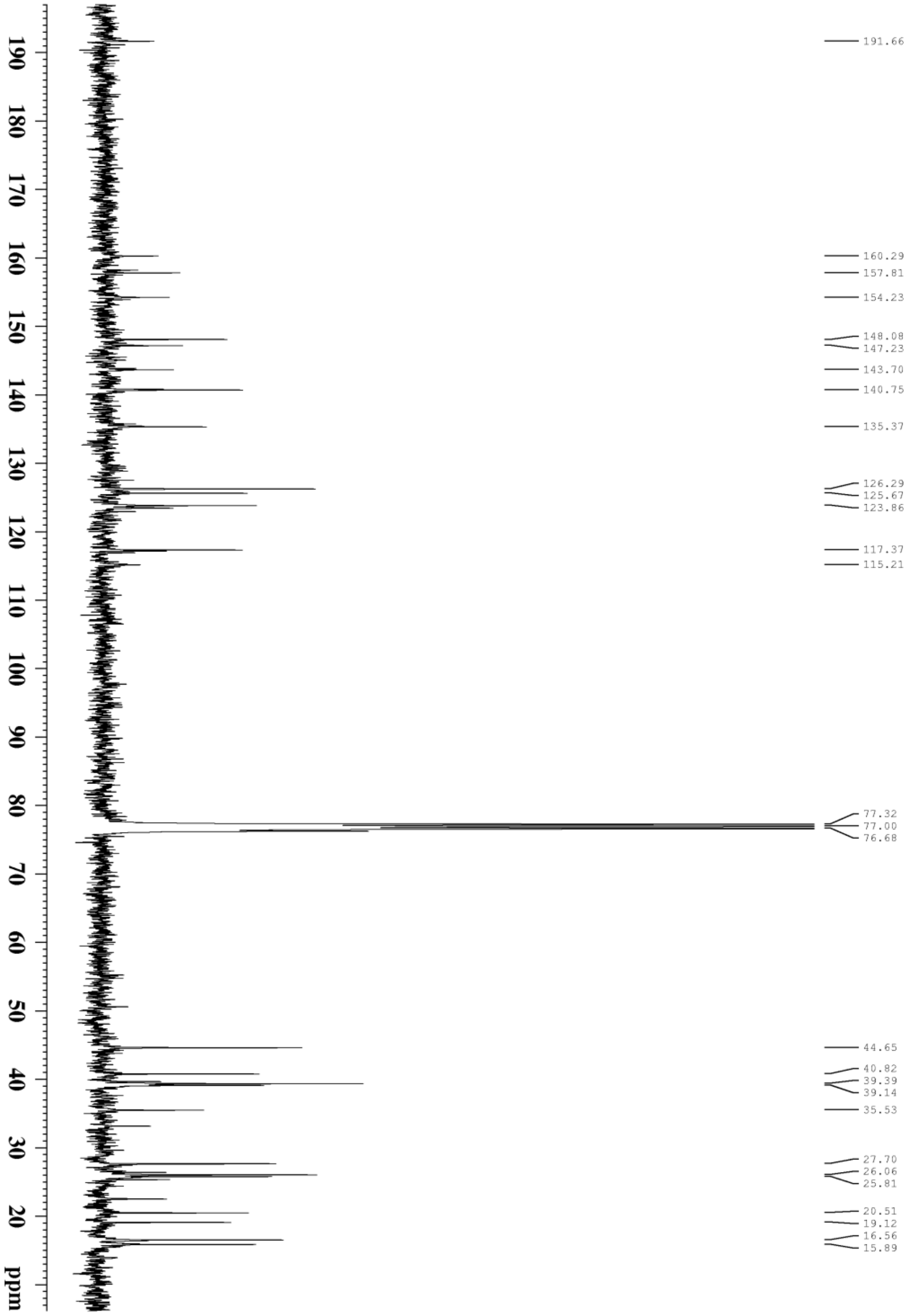

Figure S14. HSQC Spectrum of Malonganenone N (**3**).

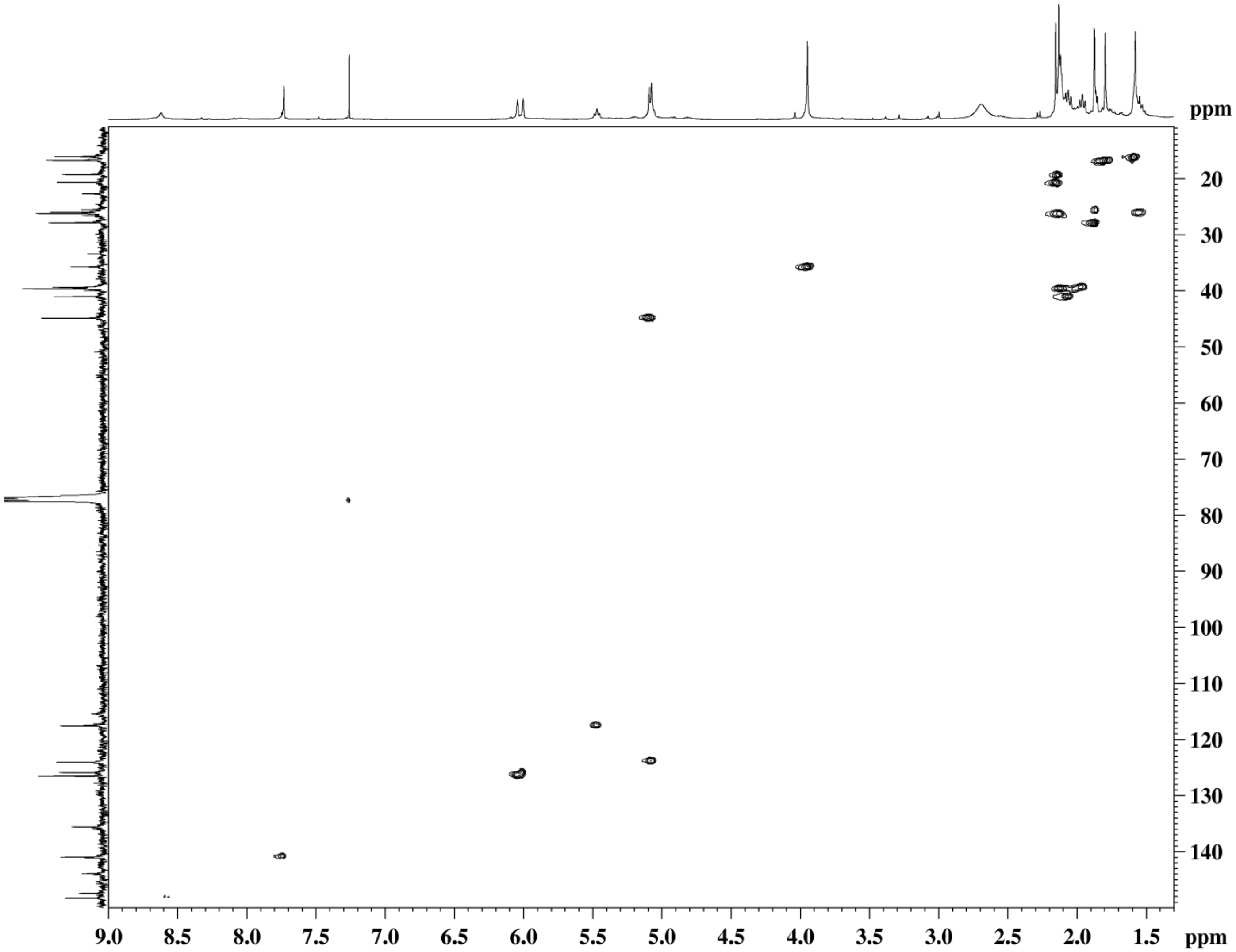

Figure S15. ^{1}H–^{1}H COSY spectrum of Malonganenone N (**3**).

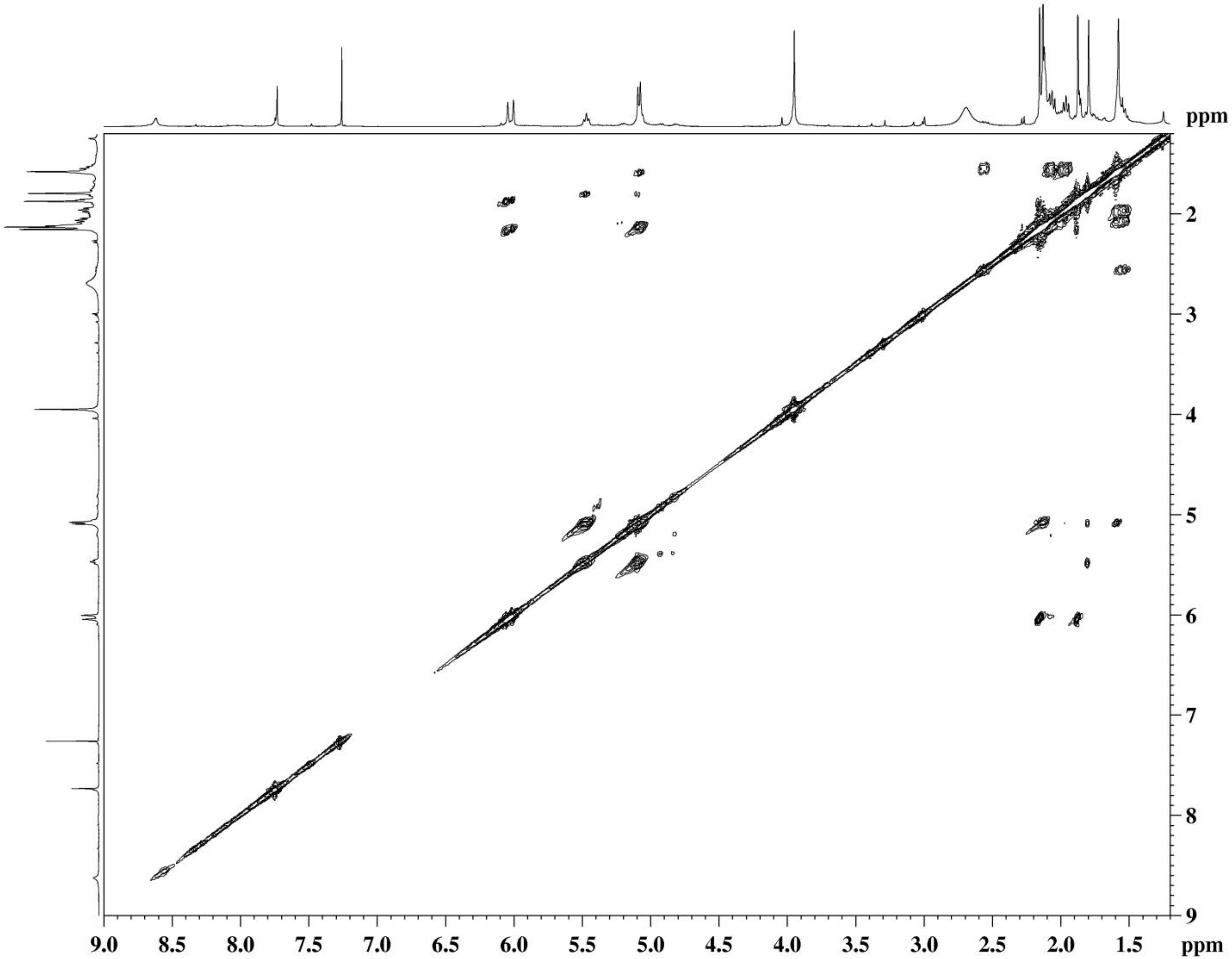

Figure S16. HMBC spectrum of Malonganenone N (**3**).

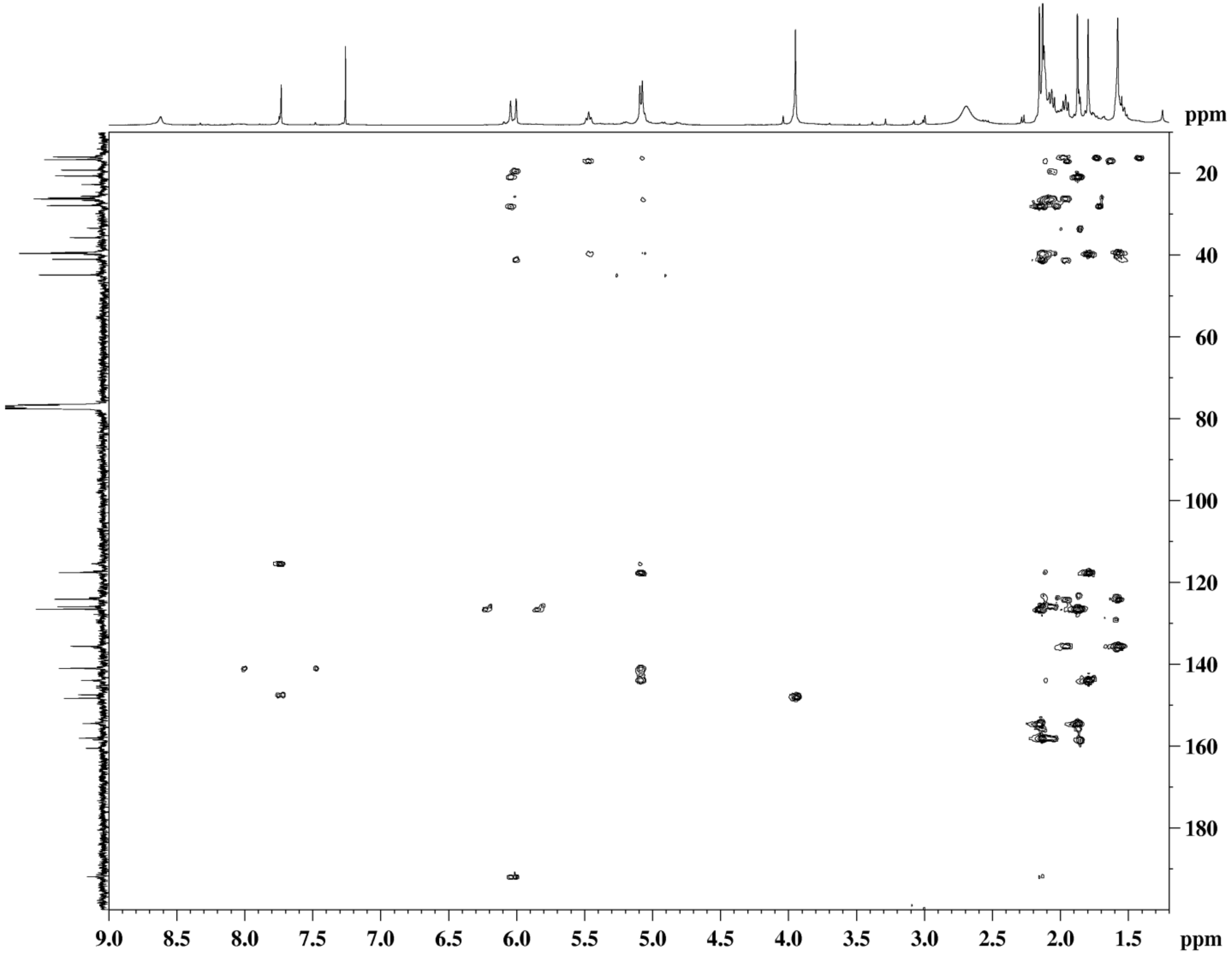

Figure S17. ^{1}H NMR spectrum of Malonganenone O (**4**).

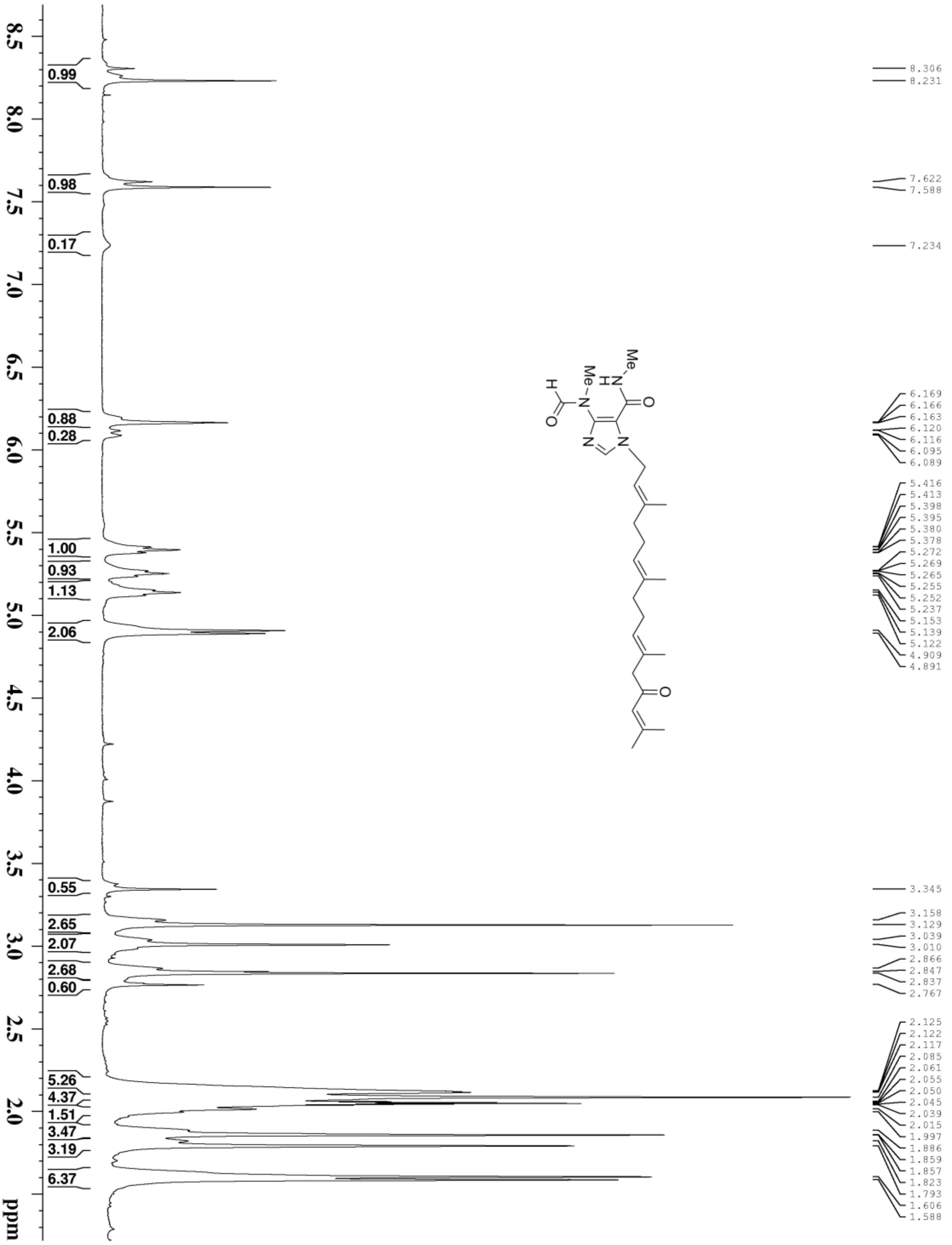

Figure S18. ^{13}C NMR spectrum of Malonganenone O (**4**).

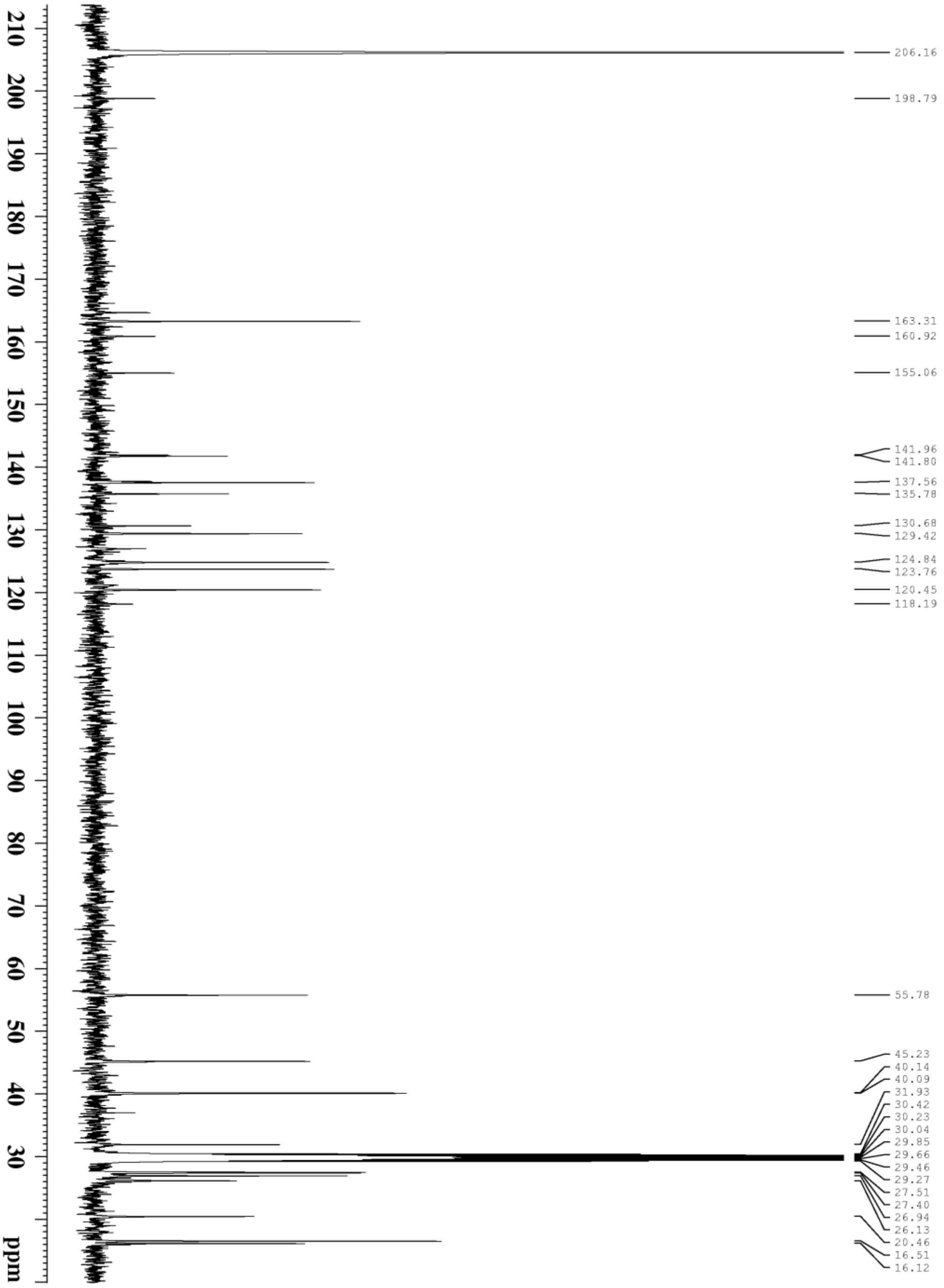

Figure S19. HSQC Spectrum of Malonganenone O (**4**).

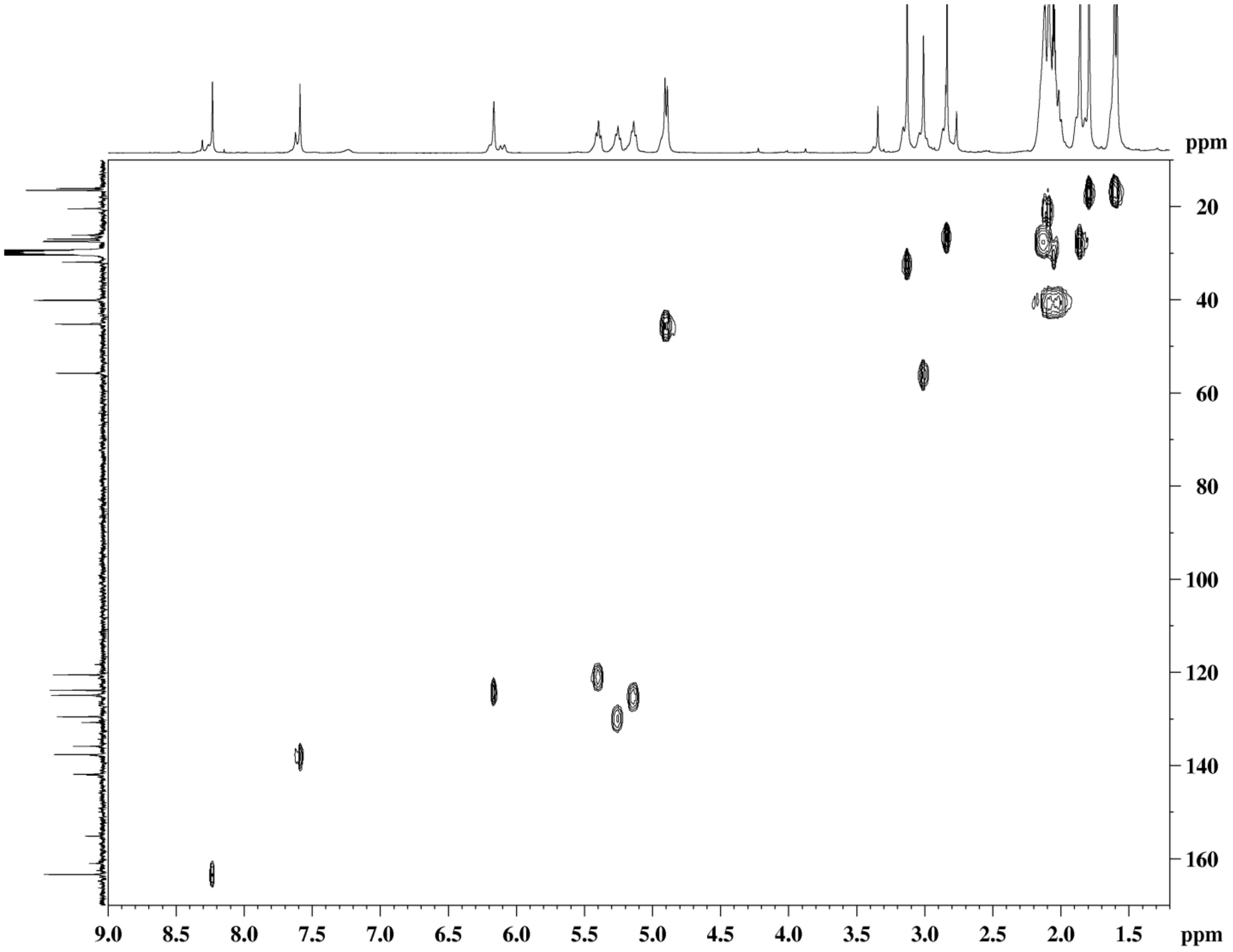

Figure S20. ^{1}H–^{1}H COSY spectrum of Malonganenone O (**4**).

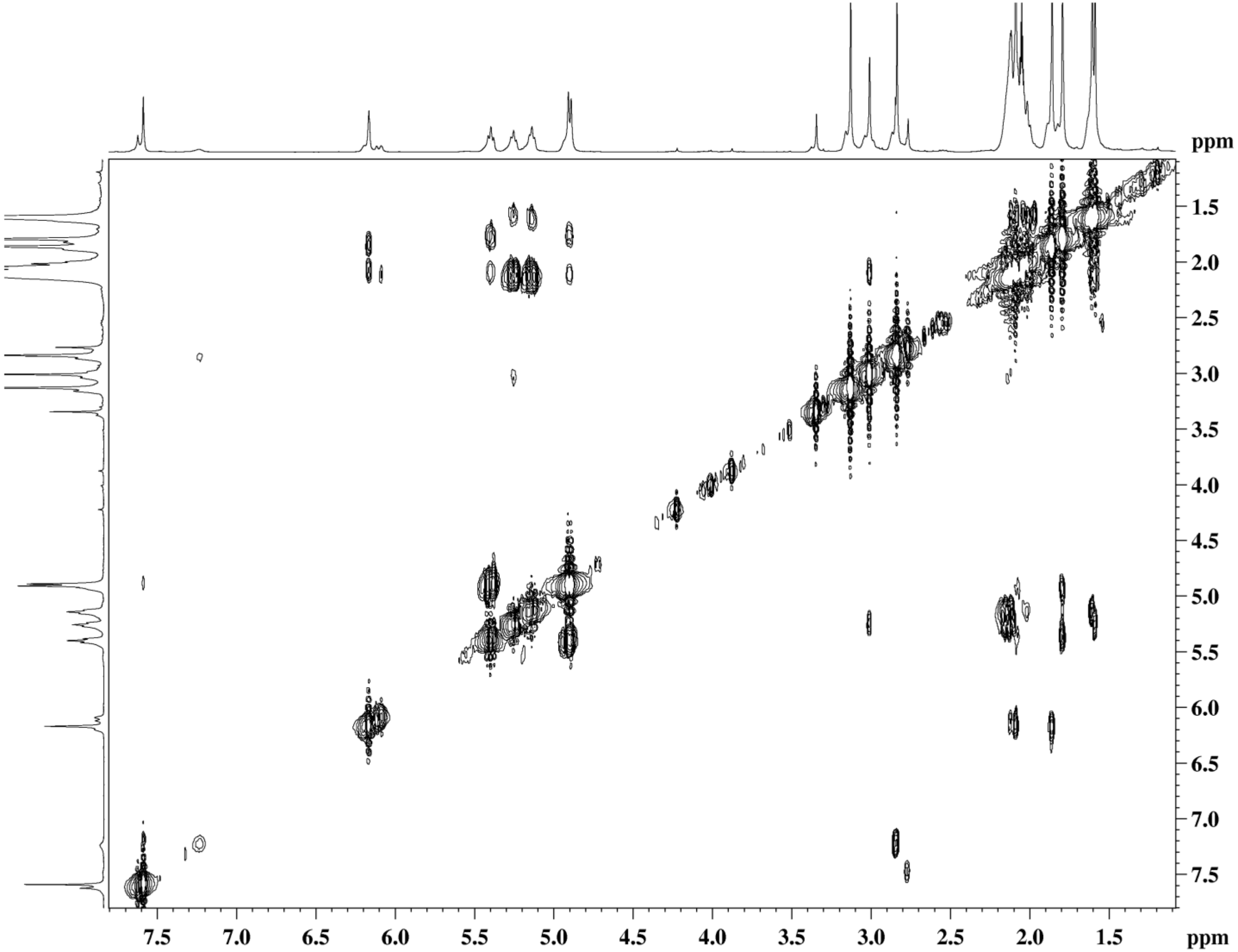

Figure S21. HMBC spectrum of Malonganenone O (**4**).

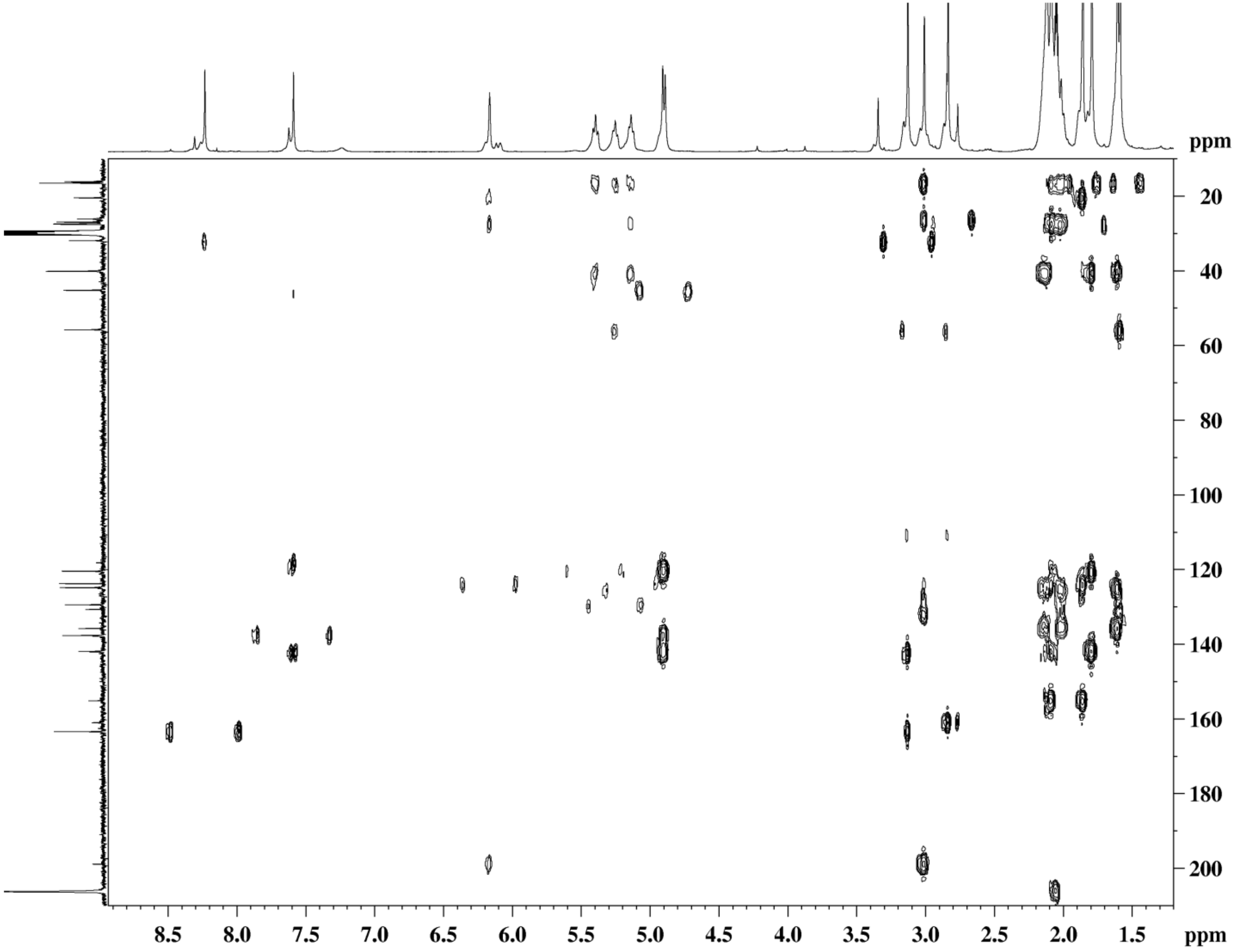

Figure S22. ^{1}H NMR spectrum of Malonganenone P (**5**).

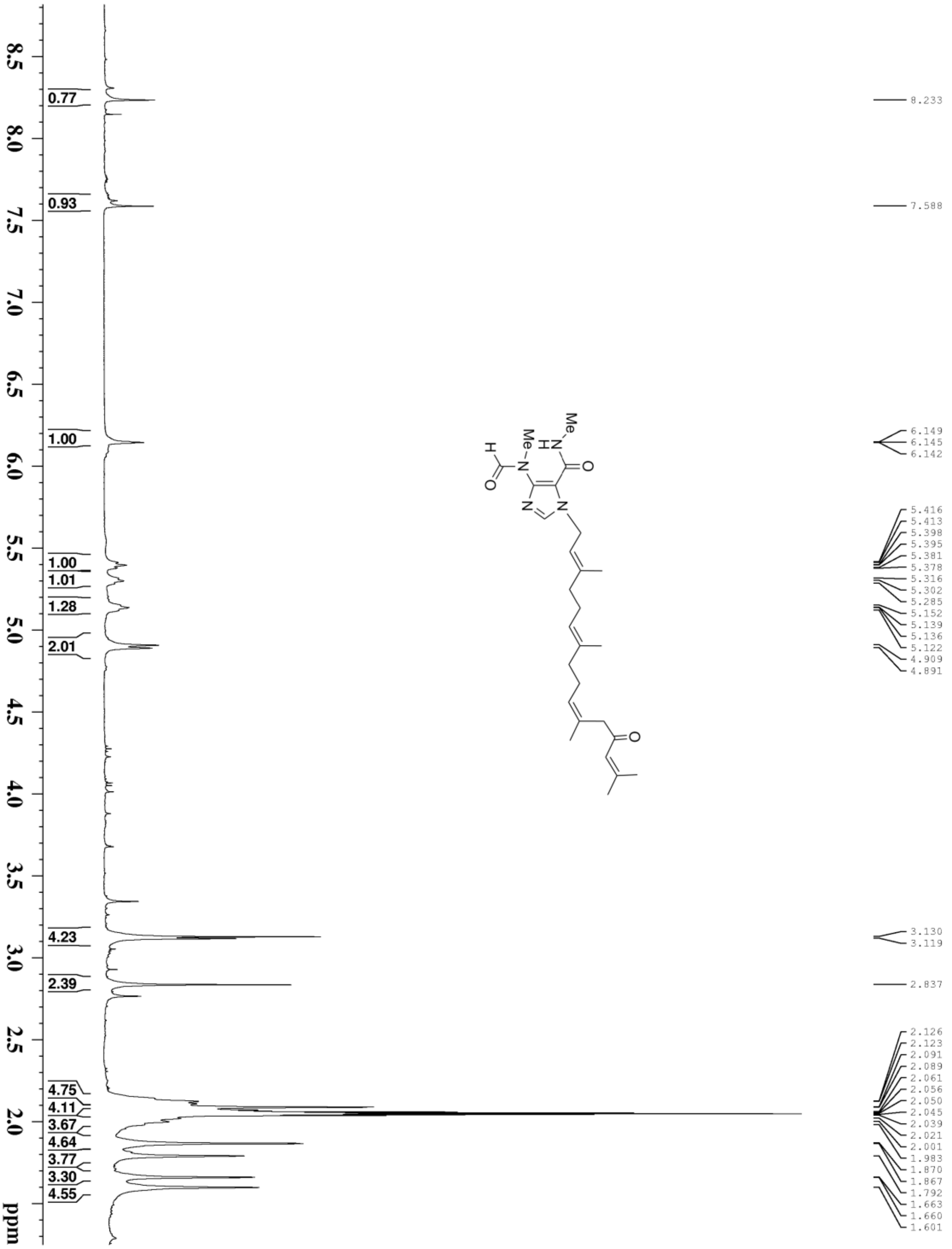

Figure S23. ^{13}C NMR spectrum of Malonganenone P (**5**).

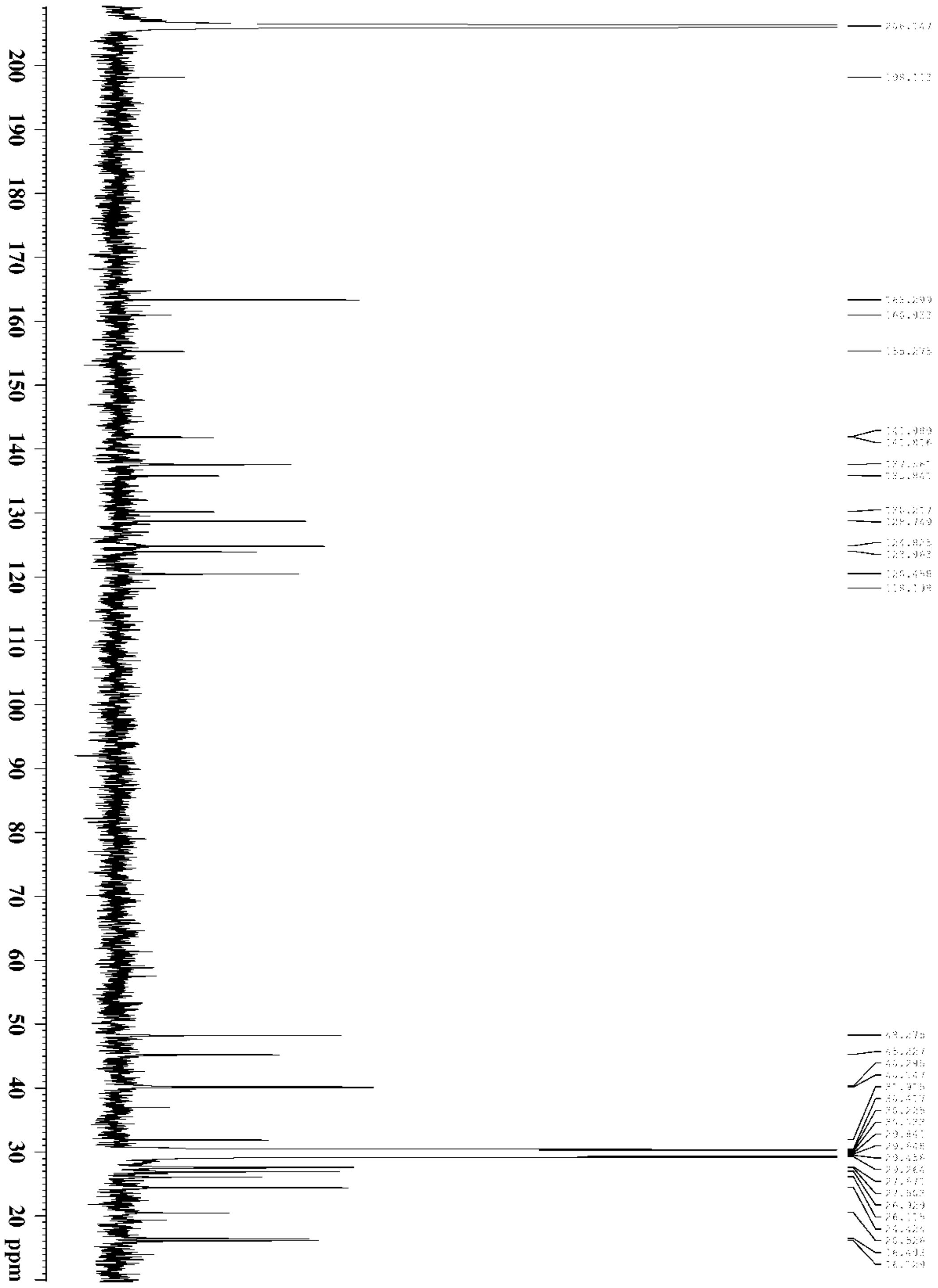

Figure S24. HSQC Spectrum of Malonganenone P (**5**).

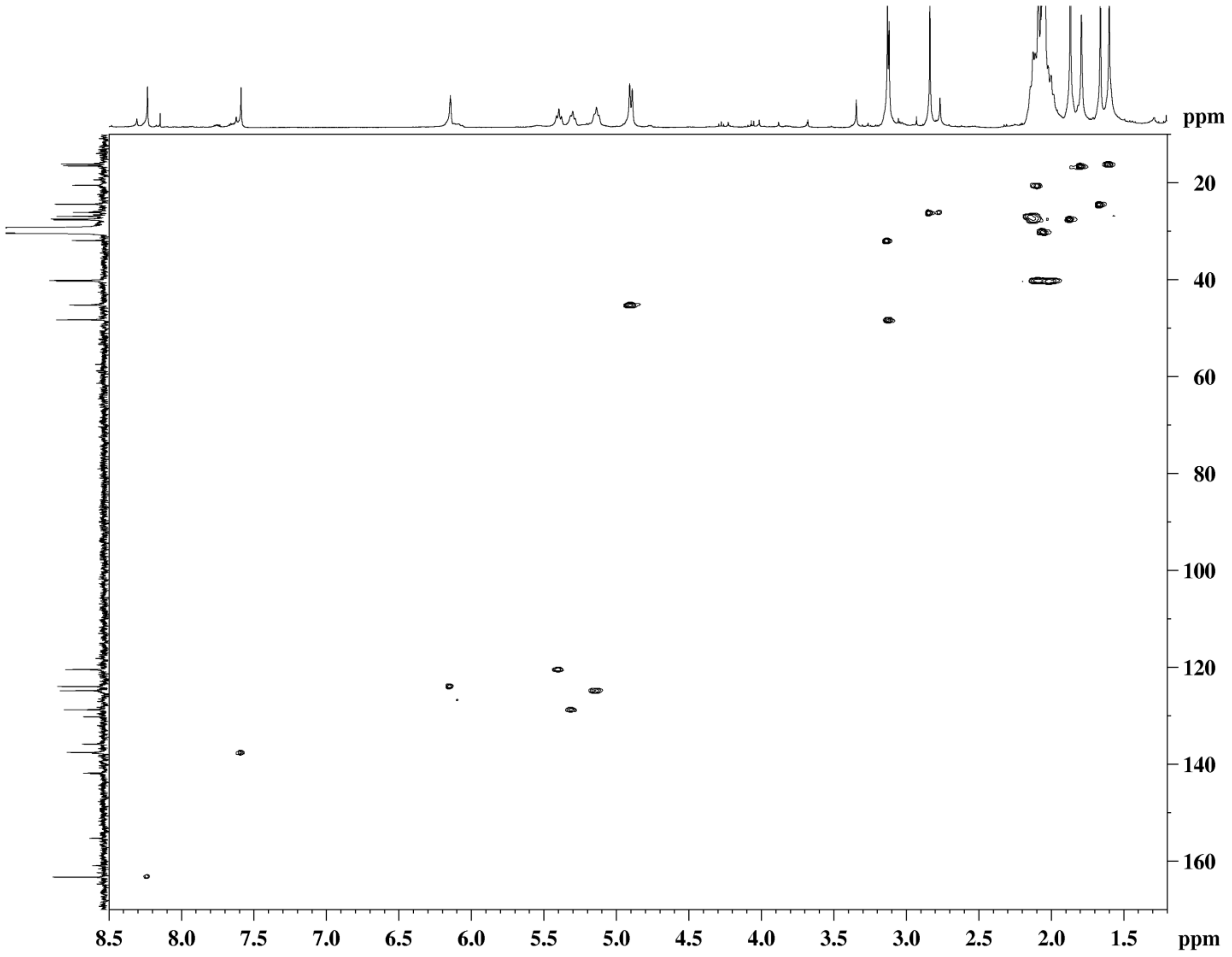

Figure S25. ^{1}H–^{1}H COSY spectrum of Malonganenone P (**5**).

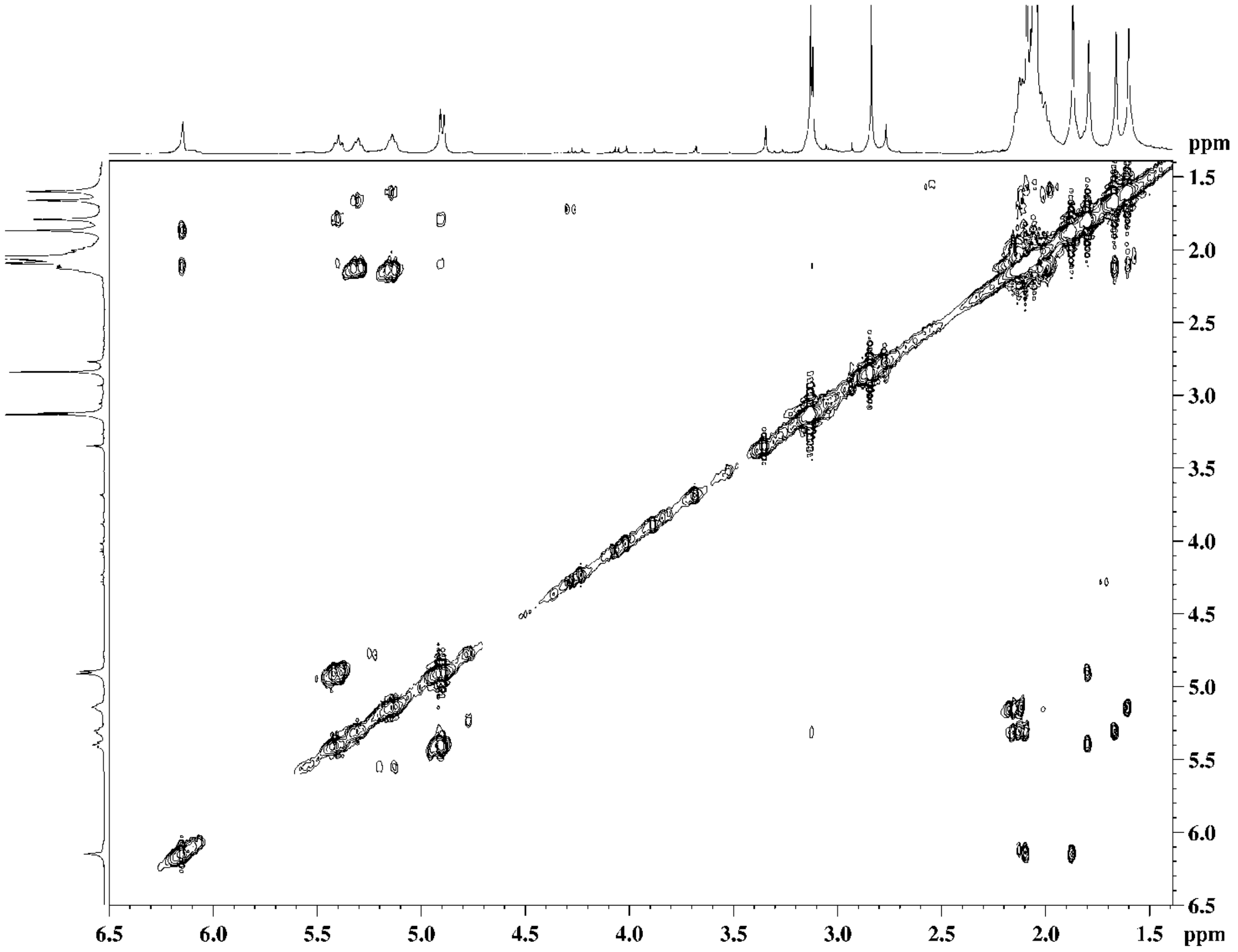

Figure S26. HMBC spectrum of Malonganenone P (**5**).

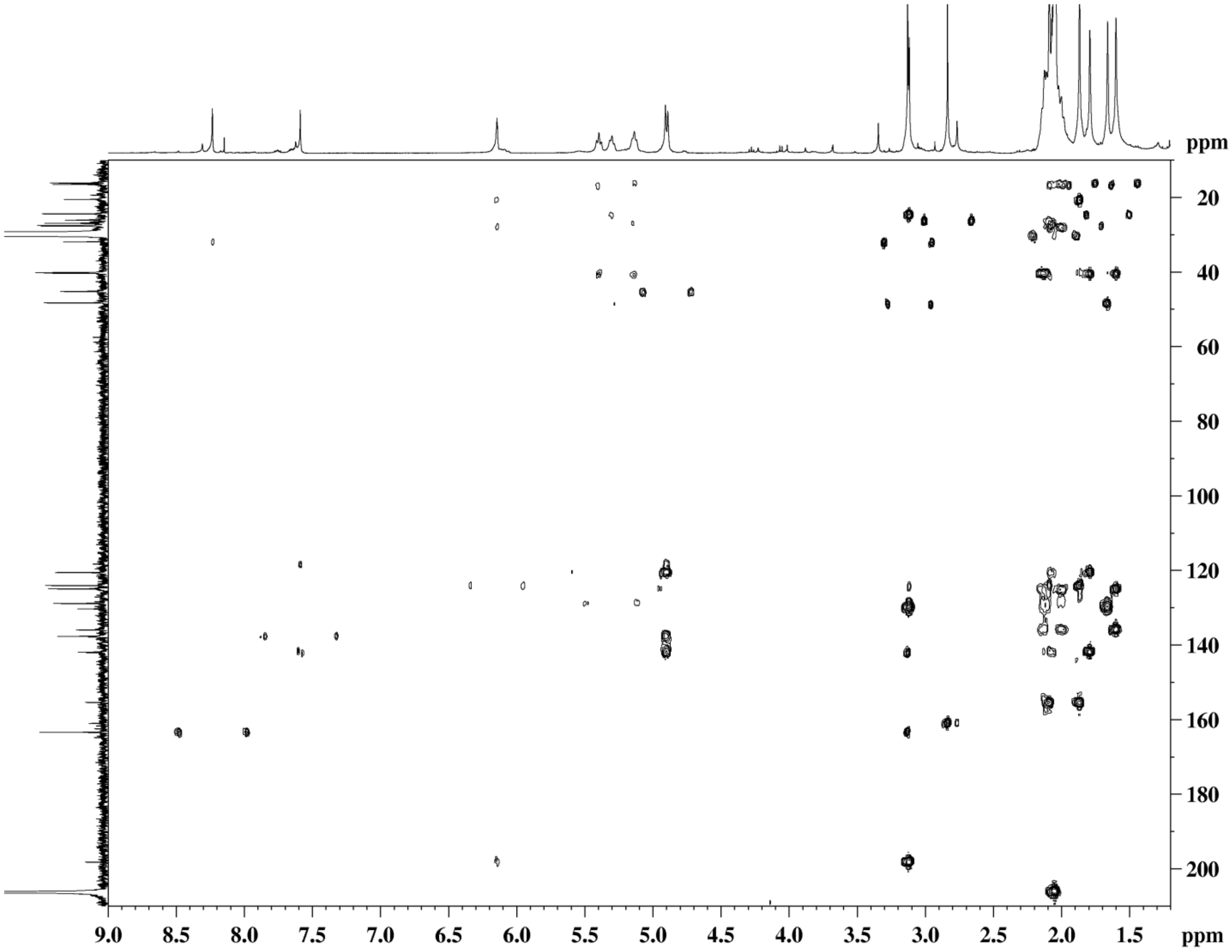

Figure S27. ^{1}H NMR spectrum of Malonganenone Q (**6**).

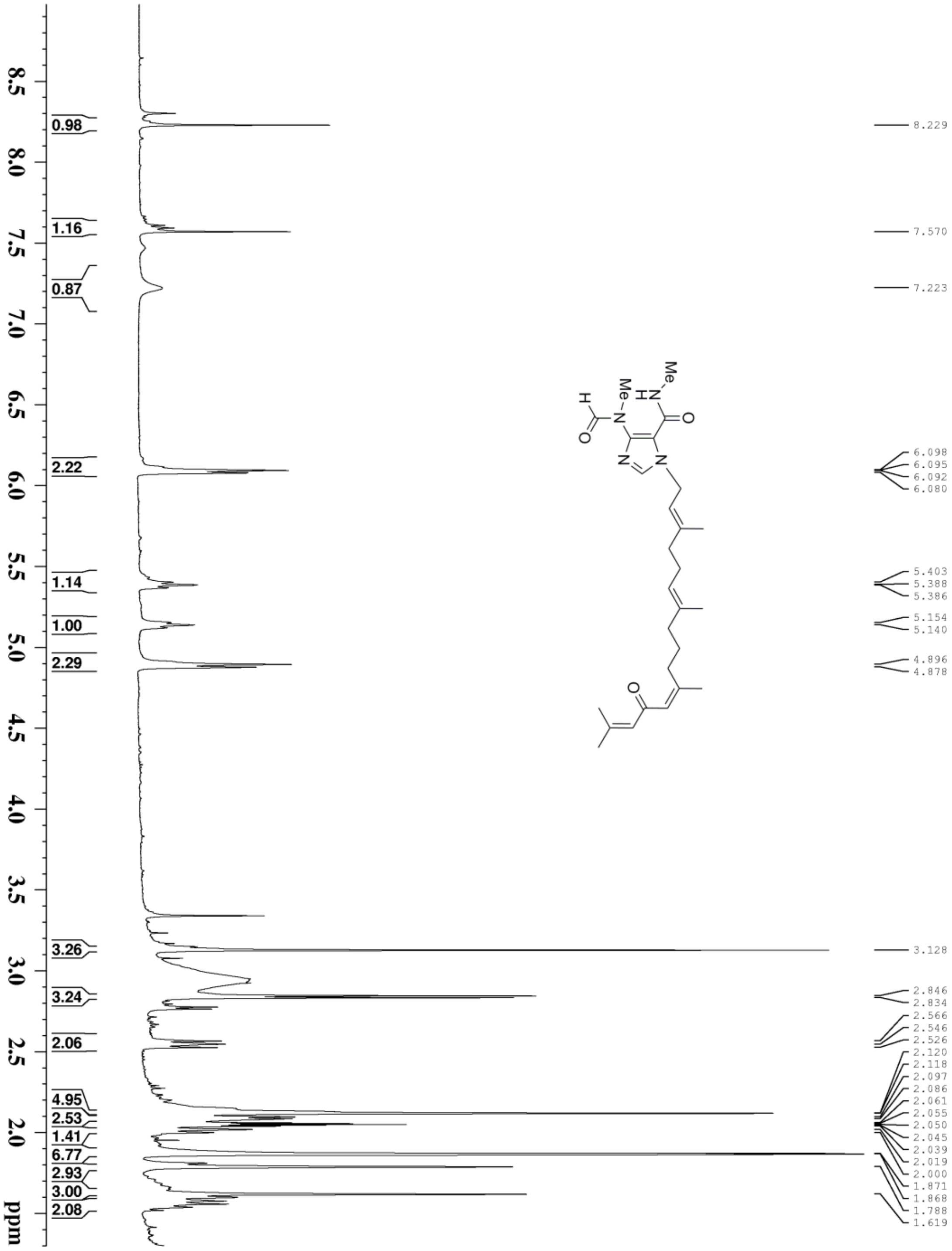

Figure S28. ^{13}C NMR spectrum of Malonganenone Q (**6**).

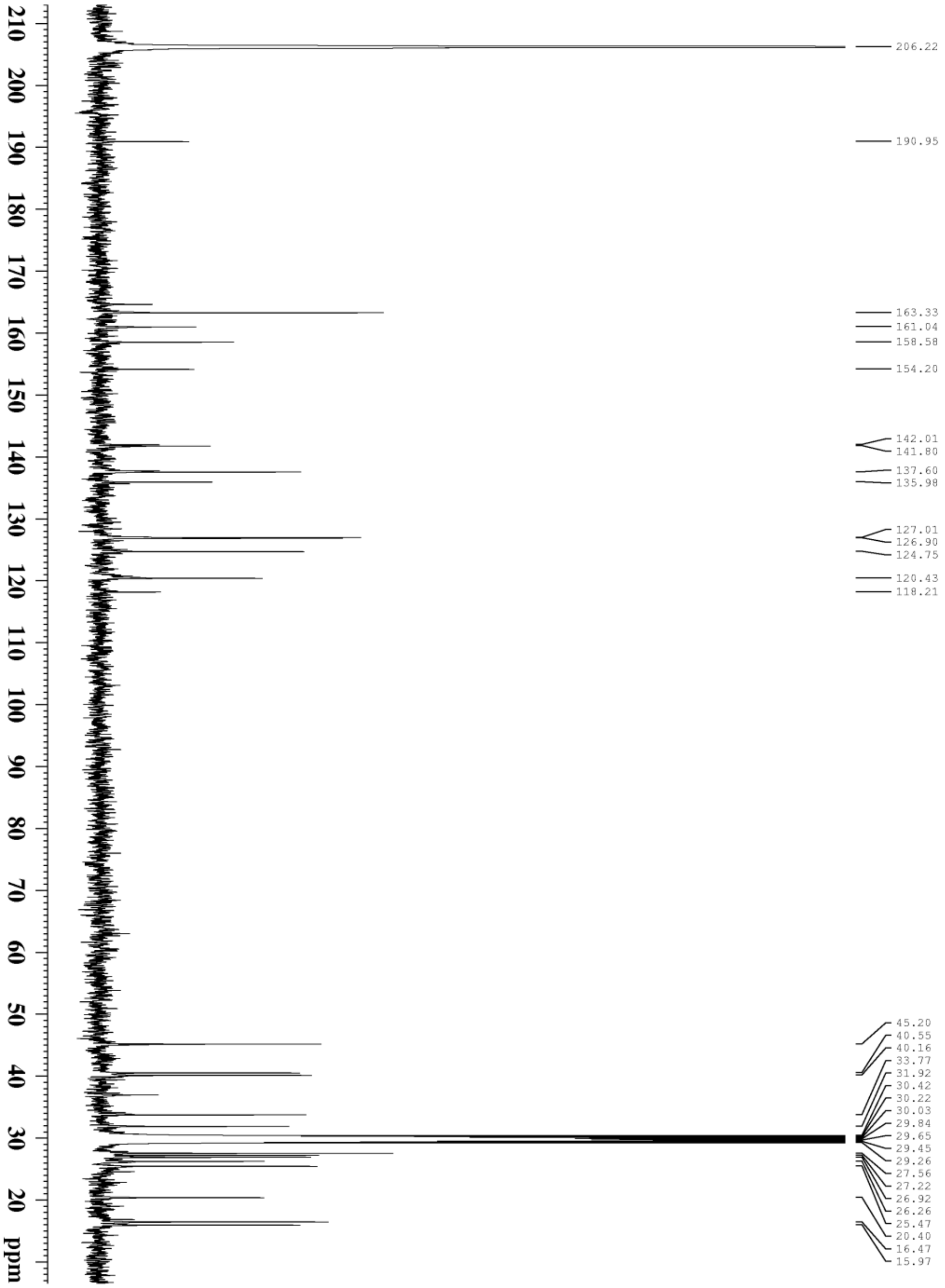

Figure S29. HSQC Spectrum of Malonganenone Q (**6**).

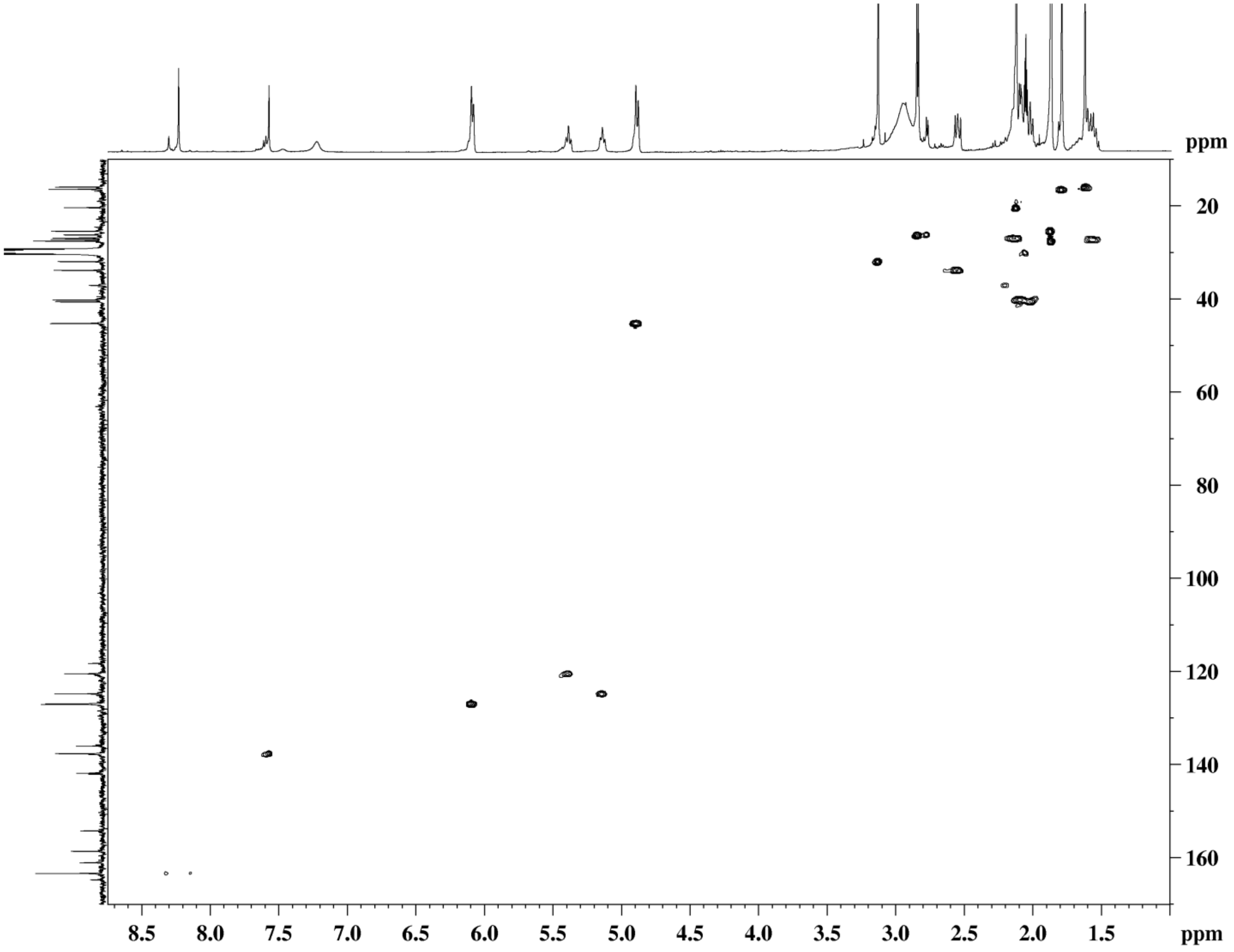

Figure S30. ^{1}H–^{1}H COSY spectrum of malonganenone Q (**6**).

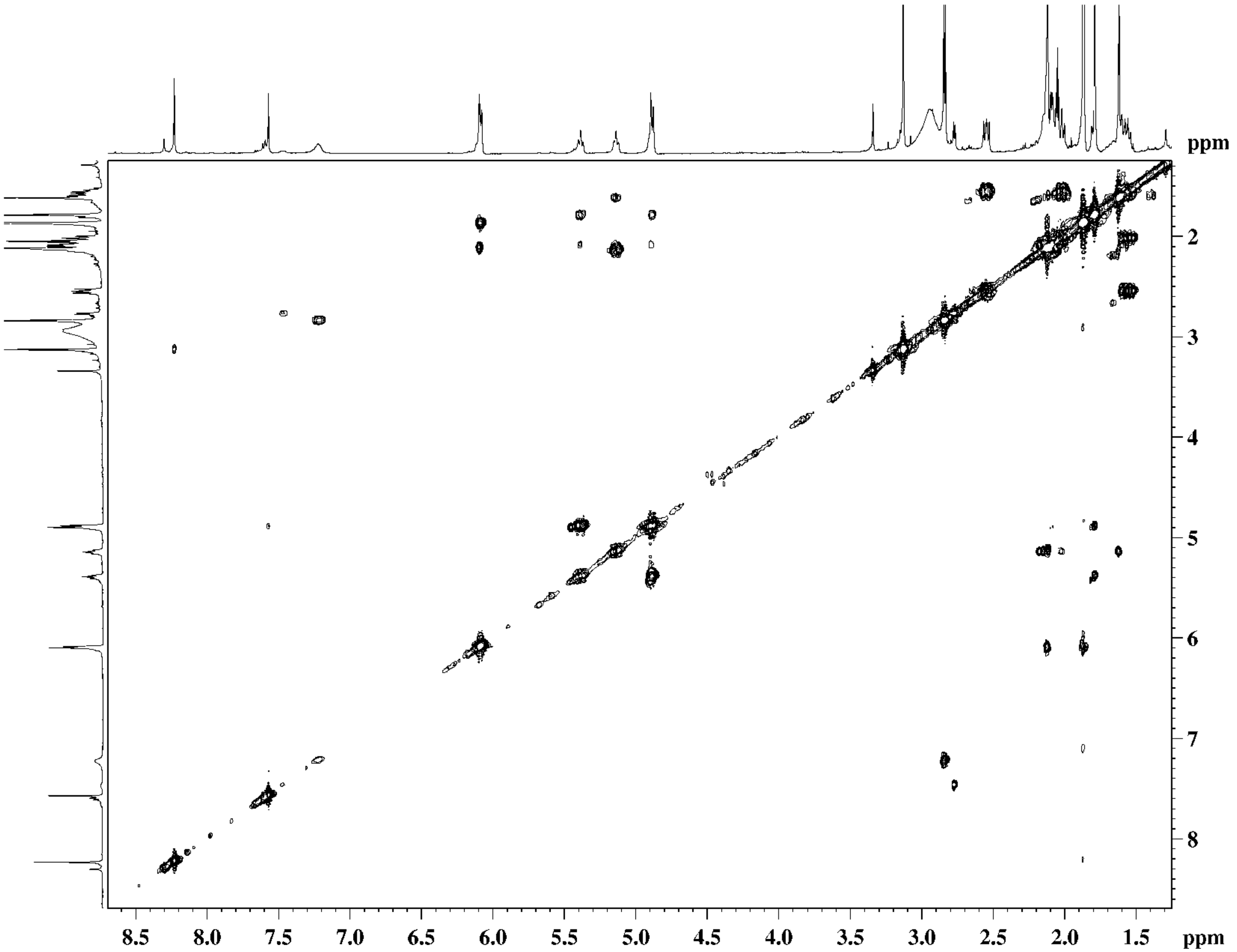

Figure S31. HMBC spectrum of malonganenone Q (**6**).

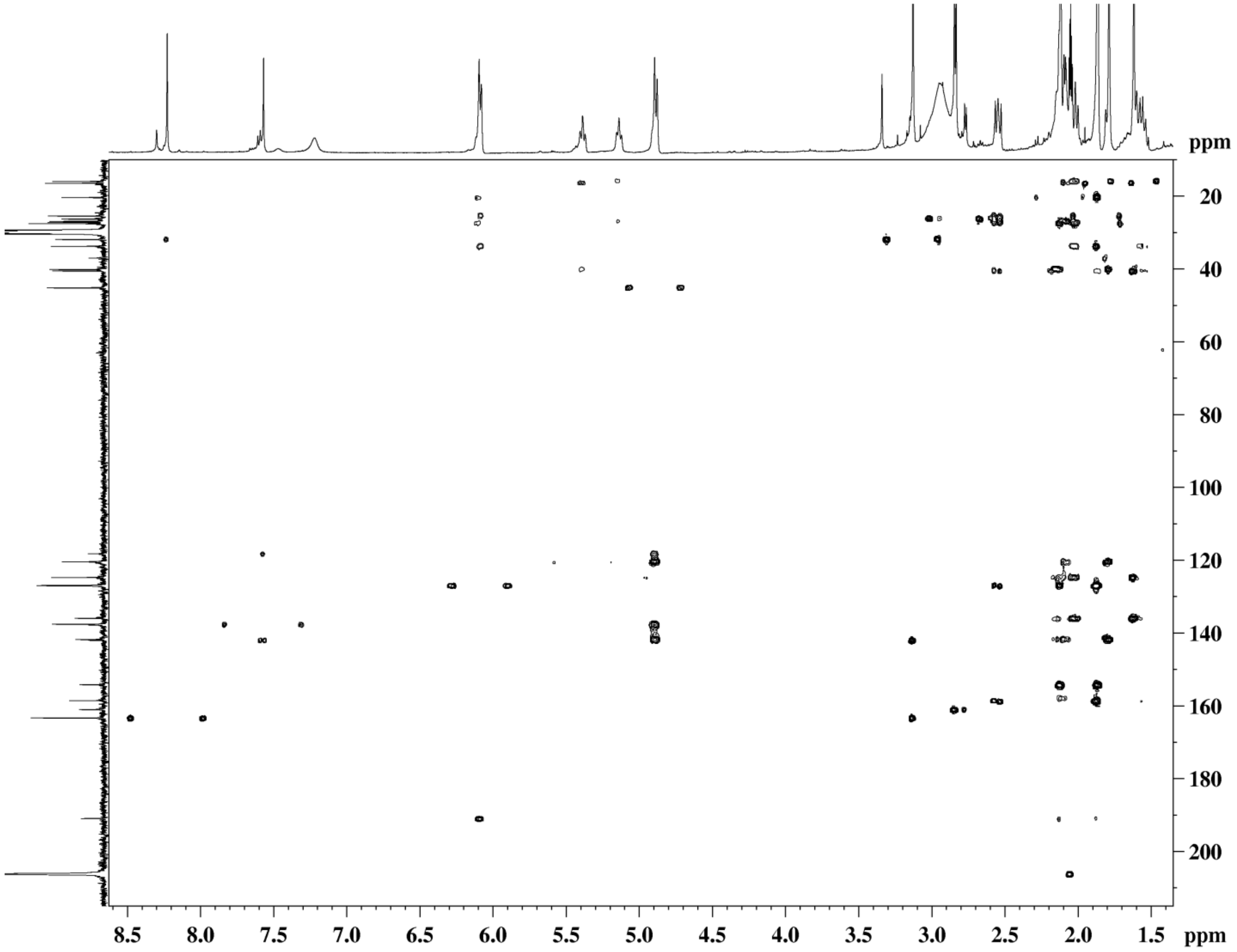

© 2014 by the authors; licensee MDPI, Basel, Switzerland. This article is an open access article distributed under the terms and conditions of the Creative Commons Attribution license (http://creativecommons.org/licenses/by/3.0/).
